# Augmenting the Calvin–Benson–Bassham cycle by a synthetic malyl-CoA-glycerate carbon fixation pathway

**DOI:** 10.1038/s41467-018-04417-z

**Published:** 2018-05-22

**Authors:** Hong Yu, Xiaoqian Li, Fabienne Duchoud, Derrick S. Chuang, James C. Liao

**Affiliations:** 1 UCLA-DOE Institute of Genomics and Proteomics, 420 Westwood Plaza, Los Angeles, CA 90095 USA; 2 Department of Chemical and Biomolecular Engineering, University of California, Los Angeles, CA 90095 USA; 3 Academia Sinica, 128 Academia Road, Section 2, 115 Taipei Taiwan

## Abstract

The Calvin–Benson–Bassham (CBB) cycle is presumably evolved for optimal synthesis of C3 sugars, but not for the production of C2 metabolite acetyl-CoA. The carbon loss in producing acetyl-CoA from decarboxylation of C3 sugar limits the maximum carbon yield of photosynthesis. Here we design a synthetic malyl-CoA-glycerate (MCG) pathway to augment the CBB cycle for efficient acetyl-CoA synthesis. This pathway converts a C3 metabolite to two acetyl-CoA by fixation of one additional CO_2_ equivalent, or assimilates glyoxylate, a photorespiration intermediate, to produce acetyl-CoA without net carbon loss. We first functionally demonstrate the design of the MCG pathway in vitro and in *Escherichia coli*. We then implement the pathway in a photosynthetic organism *Synechococcus elongates PCC7942*, and show that it increases the intracellular acetyl-CoA pool and enhances bicarbonate assimilation by roughly 2-fold. This work provides a strategy to improve carbon fixation efficiency in photosynthetic organisms.

## Abstract (web summary)

Improving carbon fixation efficiency and reducing carbon loss have been long term goals for people working on photosynthetic organism improvement. Here, the authors design a synthetic malyl-CoA-glycerate pathway for efficient acetyl-CoA synthesis and verify its function in vitro, in *E. coli* and in cyanobacterium.

## Introduction

The ribulose-1,5-bisphosphate carboxylase/oxygenase (Rubisco)-dependent CBB cycle is the most prevalent CO_2_ assimilation mechanism on Earth. The CBB cycle fixes atmospheric CO_2_ into a C3 metabolite, which serves as a precursor for all cellular constituents and most of the reduced carbon on Earth. However, the CBB cycle has its limitations. First, the CBB cycle is presumably evolved for optimal synthesis of C3 compound, but not for the production of acetyl-CoA, the C2 building block (Supplementary Table 1). When 3−phosphoglycerate (C3), the product of the CBB cycle, is converted to acetyl-CoA, one fixed carbon is lost as CO_2_ (Supplementary Fig. 1a). Second, the oxygenation reaction of Rubisco causes carbon loss during metabolism of its side product, 2-phosphoglycolate. Improvement of Rubisco specificity has been challenging^1,2^, and no natural pathway is practically feasible to convert the photorespiration intermediates, such as glycolate or glyoxylate, into acetyl-CoA without carbon loss. Furthermore, the CBB cycle involves significant ATP consumption for CO_2_ fixation (Supplementary Table 1). Since acetyl-CoA is one of the central precursor molecules involved in biosynthesis of numerous products^3–6^, its inefficient synthesis from the CBB cycle limits the maximum carbon yield of photosynthetic products and presents a major challenge for the development of bio-based economy^7,8^.

Various solutions have been proposed to reduce carbon loss during acetyl-CoA synthesis, including a synthetic non-oxidative glycolytic (NOG) pathway^8^ that can bypass the C3 decarboxylation step. The NOG pathway converts two glyceraldehyde 3-phosphate (C3) into three molecules of acetyl-phosphate (C2) without carbon loss. In plants, Rubisco shunt^9^ is evolved for carbon-conservational acetyl-CoA synthesis from sugar, which yields 20% more acetyl-CoA with 40% less carbon loss compared to glycolysis.

In contrast to Rubisco, phosphoenolpyruvate (PEP) carboxylase (Ppc) is known to be one of the most active carboxylases with no oxygenase activity^10^. The enzyme catalyzes the carboxylation of PEP (C3) to produce oxaloacetate (OAA) (C4). Ppc is used to replenish intermediates of the tricarboxylic acid (TCA) cycle for amino acid biosynthesis, or to shuttle CO_2_ between the mesophyll and bundle sheath cells in C4 plants^11^. In most organisms, however, C4 compounds cannot be metabolized to acetyl-CoA without carbon loss (Supplementary Fig. 1a)^12^. Without such a capability, carbon fixation through Ppc is of limited use.

Here we introduce a synthetic malyl-CoA-glycerate (MCG) pathway to complement the deficiency of the CBB cycle for efficient acetyl-CoA synthesis. This designed pathway is capable of converting one C3 sugar to two acetyl-CoA via fixation of one CO_2_ equivalent, or assimilating glyoxylate, a downstream product of 2-phosphoglycolate, into acetyl-CoA without net carbon loss. We first investigate the feasibility of the MCG pathway in vitro and in *Escherichia* *coli*. Then we demonstrate the effect of coupling the MCG pathway with the CBB cycle for acetyl-CoA synthesis in a photosynthetic organism *Synechococcus elongatus*.

## Results

### Design of the MCG pathway for efficient acetyl-CoA synthesis

In theory, if the NOG pathway^8^ is integrated with the CBB cycle (Supplementary Fig. 1c and d), it requires only two CO_2_ turnovers by Rubisco and six ATP to synthesize each acetyl-CoA as opposed to the endogenous route (Supplementary Fig. 1b) that needs three CO_2_ turnovers and seven ATP (Table 1). Since Rubisco is a major rate-limiting step in photosynthetic organisms, the reduced dependence on Rubisco turnover reaction is expected to improve the overall photosynthesis rate. However, overexpression of *xpk*^8^ (coding for phosphoketolase), the key gene of the NOG pathway, severely inhibited growth of *Synechococcus elongatus* (Supplementary Fig. 1e and f). Since both pathways compete for the same intermediates (Supplementary Fig. 1b), the integration of the NOG pathway with the CBB cycle was not readily feasible.

**Table 1:** Comparison of different pathway combinations for synthesizing each acetyl-CoA from CO_2_ equivalents

Per Ac-CoA synthesis from CO_2_	NAD(P)H consumption	ATP consumption	Rubisco turnover	The theoretical carbon yield
CBB + PDH^a^	4	7	3	66% (1 Ac-CoA/C3)
CBB + NOG	4	6	2	100% (1.5 Ac-CoA/C3)
CBB + rGS–citrate	4	5	1.5	100% (2 Ac-CoA/C3 + C1)
CBB + MCG	4	5.5	1.5	100% (2 Ac-CoA/C3 + C1)

^a^PDH: pyruvate dehydrogenase complex; CBB + PDH is the native pathway

**Fig. 1:**
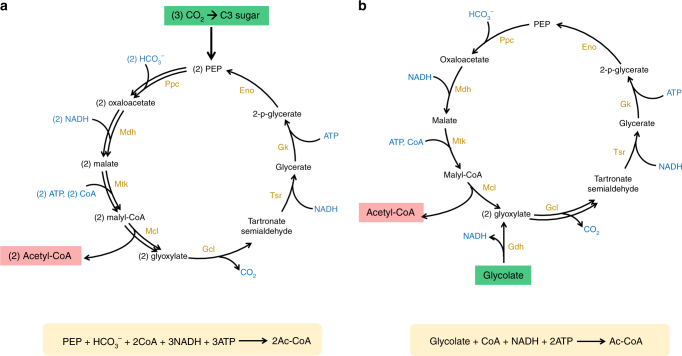
Design of the MCG pathway for efficient acetyl-CoA synthesis. **a** The MCG pathway can convert one C3 sugar to two acetyl-CoA via fixation of one CO_2_ equivalent. PEP, phosphoenolpyruvate; Ac-CoA, acetyl-CoA; Ppc, phosphoenolpyruvate carboxylase; Mdh, malate dehydrogenase; Mtk, malate thiokinase; Mcl, malyl-CoA lyase; Gcl, glyoxylate carboligase; Tsr, tartronate semialdehyde reductase; Gk, glycerate kinase; Eno, enolase. **b** The MCG pathway, coupling with glycolate dehydrogenase, can assimilate glycolate to acetyl-CoA without net carbon loss. Gdh, glycolate dehydrogenase. The net reactions are shown in the yellow boxes

We thus designed two other synthetic pathways, termed the reverse glyoxylate shunt-citrate (rGS–citrate) pathway (Supplementary Fig. 2a) and the MCG pathway (Fig. 1a), to couple with the CBB cycle. These two pathways do not share the same intermediates with the CBB cycle, and both of them are more efficient in acetyl-CoA synthesis compared to the NOG. These pathways can convert one PEP (C3) to generate two acetyl-CoA via fixation of one CO_2_ equivalent (Table 1). We showed the feasibility of part of the rGS–citrate pathway in an oxaloacetate auxotrophic *E*. *coli* strain^13^ (Supplementary Fig. 2a). However, we were unable to demonstrate the complete rGS–citrate pathway, possibly due to its non-robustness predicted by computational analysis^14^. The metabolic activities from malate to glyoxylate and to succinate need to be balanced in order to maintain equal flux. Otherwise, imbalanced flux would cause accumulation or depletion of pathway intermediates, and ultimately stop the rGS–citrate pathway.

We then focused on the MCG pathway. In this pathway (Fig. 1a and Supplementary Table 2), an input PEP together with a regenerated PEP are carboxylated to produce two oxaloacetate via assimilation of two bicarbonate. Two oxaloacetate molecules are reduced to malate, which is activated to malyl-CoA, and further split into two acetyl-CoA and two glyoxylate. The two acetyl-CoA are the products of this pathway, and two glyoxylate are recycled to regenerate one PEP through a bacterial glyoxylate assimilation route. To do so, two glyoxylate are condensed to one tartronate semialdehyde (C3), releasing one CO_2_, through glyoxylate carboligase (Gcl). Then tartronate semialdehyde is reduced to d-glycerate and phosphorylated to form 2-phosphoglycerate by tartronate semialdehyde reductase (Tsr) and glycerate kinase (Gk), respectively. Thus, the net reaction of the MCG pathway is to convert one PEP and one bicarbonate to produce two acetyl-CoA with the expense of three ATP and three NADH (Fig. 1a). If the pathway is constructed in a photosynthetic organism (Supplementary Fig. 2c), the cell will need only 1.5 CO_2_ assimilation by Rubisco to produce one acetyl-CoA with the expenditure of 5.5 ATP and 4 NADH (Table 1). This is a significant improvement over the native system.

Meanwhile, the MCG pathway can also assimilate C2 metabolites, such as photorespiration intermediates glycolate and glyoxylate (Fig. 1b), to acetyl-CoA with 100% theoretical carbon efficiency. This capability is particularly useful for C3 plants which suffer from severe carbon loss by photorespiration under hot dry conditions^15,16^. The net reactions of converting glycolate to acetyl-CoA by various pathways are compared in Table 2. To our knowledge, no natural pathway can perform the complete carbon conversion from glycolate to acetyl-CoA, and the synthetic MCG pathway is the only one with such type of activity.

**Table 2:** Comparison of ATP/NADH consumption and carbon yield among different pathways in assimilation of glycolate to produce acetyl-CoA

Per Ac-CoA synthesis from glycolate	NAD(P)H consumption	ATP consumption	The theoretical carbon yield
Native photorespiration pathway^41^	0	0	50% (1 Ac-CoA/2 glycolate)
The bacterial glycolate assimilation route^41^	−2	0	50% (1 Ac-CoA/2 glycolate)
The MCG pathway	1	2	100% (1 Ac-CoA/1 glycolate)

The MCG pathway can convert one glycolate to produce one acetyl-CoA without net carbon loss. The bacterial glycolate assimilation route converts two glycolate to only one acetyl-CoA

### Establishing an in vivo platform for Mtk/Mcl activity test

**Fig. 2:**
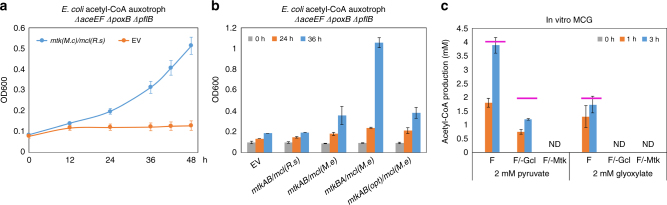
Rescue of an acetyl-CoA auxotroph and demonstration of the MCG pathway in vitro. **a** Expression of *mtk*(*M.c*)*/mcl*(*R.s*) could rescue the growth defect of the acetyl-CoA auxotroph (*∆aceEF ∆poxB ∆pflB*) and allowed the strain to grow in minimal medium with 1% glucose addition within 48 h. EV, empty vector. **b** Expression of the genes in the order of *mtkB*(*M.c*)*/mtkA*(*M.c*)*/mcl*(*M.e*) improved the growth of the *∆aceEF ∆poxB ∆pflB* strain to the highest culture density in minimal medium with glucose as the sole carbon source in 36 h. Opt, codon optimization. **c** Measurement of acetyl-CoA production through the in vitro MCG pathway. Two millimolar pyruvate (or glyoxylate) was used as the initial substrate to evaluate the effect of the pathway. F, full enzyme mixture; F/-Gcl, full enzyme mixture omitting Gcl; F/-Mtk, full enzyme mixture without Mtk addition; ND, not detectable. Magenta lines indicated the theoretical titer of the MCG pathway by using 2 mM pyruvate (or glyoxylate) as the initial substrate. Error bars are s.d. (standard deviation), *n* = 3

The most important step of the MCG pathway is to split malate to produce acetyl-CoA and glyoxylate, catalyzed by malate thiokinase (Mtk) and malyl-CoA lyase (Mcl). Therefore, we designed an in vivo platform to screen for Mtk and Mcl. We constructed an acetyl-CoA auxotrophic strain of *E*. *coli* by deleting all the genes (*pflB*^17^, *poxB*^18^, and *aceEF*^19^) that code for enzymes producing acetyl-CoA from pyruvate (Supplementary Fig. 3a). Such a strain cannot grow in minimal medium with glucose as the sole carbon source unless supplemented with acetate (Supplementary Fig. 3a). We showed that expression of *mtk* from *Methylococcus capsulatus* and *mcl* from *Rhodobacter sphaeroides* could rescue the growth defect of the acetyl-CoA auxotroph (*∆aceEF ∆poxB ∆pflB*) and allowed the *E*. *coli* strain to grow in minimal medium with only glucose addition (Fig. 2a), which suggested Mtk(*M*.*c*) together with Mcl(*R*.*s*) split malate to generate acetyl-CoA for growth-supporting. Thus, we used this *E*. *coli* system to screen for a suitable Mtk/Mcl combination.

The results showed Mtk (originally annotated as SucCD-2) from *M*. *capsulatus* was still the most active enzyme to convert malate to malyl-CoA. However, a more active Mcl (MexAM1_META1p1733) from *Methylobacterium extorquens* was found (Fig. 2b). The specific activity of purified Mcl(*M*.*e*) was nine-fold higher than the one from *R*. *sphaeroides* previously used (Supplementary Table 4). Expression of the genes in the order of *mtkB*(*M*.*c*)/*mtkA*(*M*.*c*)/*mcl*(*M*.*e*) allowed the acetyl-CoA auxotrophic strain to grow to the highest culture density within 36 h compared to other combinations (Fig. 2b). Codon optimization of *mtk*(*M*.*c*) did not improve the growth. Distinct growth-rescuing effects of *E*. *coli* strains shown in Fig. 2b were supported by their expressed Mtk/Mcl activities (Supplementary Fig. 3b and Supplementary Table 3). All of these results indicated that expression of *mtkBA*(*M*.*c*)/*mcl*(*M*.*e*) exhibited higher activity in splitting malate to produce acetyl-CoA and glyoxylate in vivo.

### Demonstration of the feasibility of the MCG pathway in vitro

Some synthetic pathways, such as the rGS–citrate pathway (Supplementary Fig. 2a), designed based on stoichiometry and thermodynamics, may be difficult or impossible to realize in vivo because of the lack of kinetic robustness^14^. For example, a narrow range of enzyme activity ratio may need to be satisfied in order to distribute the flux precisely for the cycle. To test if the MCG pathway can be readily balanced, we first set up an in vitro system (Supplementary Note 1, Supplementary Fig. 4 and Supplementary Table 4) to investigate the kinetic feasibility of the pathway by using pyruvate (C3) or glyoxylate (C2) as an initial substrate. Pyruvate, which can be phosphorylated to PEP by Pps (PEP synthase), is the direct source for acetyl-CoA synthesis in nature. On the other hand, using glyoxylate as the substrate can evaluate the capability of the MCG pathway to assimilate glycolate to produce acetyl-CoA.

The results showed that after 3 h, the substrate, 2 mM pyruvate (or glyoxylate), was completely consumed. About 3.8 mM acetyl-CoA was produced with the complete pathway enzymes using pyruvate as the initial carbon input (Fig. 2c). While as controls, only 1.2 mM acetyl-CoA was detected in the mixture without Gcl addition through the action of Mtk/Mcl, and no acetyl-CoA was produced in the mixture without Mtk. The acetyl-CoA/pyruvate molar ratio was 1.91 using the complete pathway, reaching 95% of the theoretical value (=2). The lack of complete conversion is presumably due to intermediates accumulating in the system. However, when Gcl was absent, the acetyl-CoA/pyruvate ratio was 0.6, representing only 60% of the theoretical value (=1), which was presumably caused by the inhibited Mcl (*M*.*e*) activity resulting from glyoxylate accumulation^13^.

When 2 mM glyoxylate was used as the substrate, 1.7 mM acetyl-CoA was produced from the complete pathway mixture, and no acetyl-CoA was found in the mixture without either Gcl or Mtk (Fig. 2c). The acetyl-CoA/glyoxylate molar ratio was 0.86, achieving 86% of the theoretical yield (=1), which indicated the efficiency of the glyoxylate recycling branch of the pathway. These results demonstrated the in vitro biochemical and kinetic feasibility of using the MCG pathway for acetyl-CoA synthesis.

### Construction of the MCG pathway in *E*. *coli*

To demonstrate its feasibility in vivo, we first constructed the MCG pathway in *E*. *coli*. We deleted the *gcl* gene in the acetyl-CoA auxotroph (*∆aceEF ∆poxB ∆pflB*) in order to determine whether the first segment of the MCG pathway could rescue the growth defect of the *∆aceEF ∆poxB ∆pflB ∆gcl* strain without recycling glyoxylate. It showed the expression of *mtk*(*M*.*c*)/*mcl*(*R*.*s*) was indeed able to support the *∆aceEF ∆poxB ∆pflB ∆gcl* strain to grow in minimal medium with glucose as the sole carbon source after 72 h (doubling time of 3.7 h) (Supplementary Fig. 5a). Additional overexpression of *gcl*(*E*.*c*) could accelerate the cell growth (doubling time of 3.3 h), suggesting that glyoxylate recycling was beneficial, even though the steps from tartronate semialdehyde to 2-phosphoglycerate were catalyzed by un-augmented native enzymes (Supplementary Fig. 5b). The reason to use Mcl(*R*.*s*), rather than the more active Mcl(*M*.*e*) in the experiment, was to enlarge the effect of *gcl*(*E*.*c*) overexpression.

We next constructed a pyruvate auxotroph of *E*. *coli* by deleting the enzymes (MaeA^20,21^, MaeB, and Pck^22^) that catalyze the C4 decarboxylation to C3 compound (Supplementary Fig. 3c). This strain (*∆maeAB ∆pck*) cannot grow in minimal medium with C4 or C2 compound, such as aspartate or acetate, as the sole carbon source, but can grow on pyruvate or its upstream sugars (Supplementary Fig. 3c). Expression of *mtk*(*M*.*c*)/*mcl*(*M*.*e*) could rescue the growth defect of *∆maeAB ∆pck* and allowed the strain to grow in minimal medium with aspartate as the sole carbon source (doubling time of 3.8 h) (Supplementary Fig. 5c). However, with an additional *gcl* knockout in the pyruvate auxotrophic strain, no growth-rescuing was observed by *mtk/mcl* expression within 6 days. Such results demonstrated the critical role of Gcl for PEP regeneration in the MCG pathway (Supplementary Fig. 5d).

Two glyoxylate assimilation routes can be used to catalyze the conversion of tartronate seminaldehyde to glycerate in *E*. *coli* (Supplementary Fig. 5f). One uses GlxR (or GarR), functioning as tartronate seminaldehyde reductases to directly reduce tartronate seminaldehyde to form glycerate. The second route adopts Hyi (hydroxypyruvate isomerase) and GhrA (or GhrB) (hydroxypyruvate reductase). Here tartronate semialdehyde is first converted to hydroxypyruvate and then reduced to glycerate. To investigate which metabolic route works better in *E. coli*, enzymes in these two routes were overexpressed in the wild-type strain *BW25113*. The results showed that overexpression of *gcl*(*E*.*c*)/*hyi*(*E.c*) led the strain to grow in minimal medium with 50 mM glyoxylate as the sole carbon source (doubling time of 3.1 h), while expressing either *gcl*(*E*.*c*)/*glxR*(*E.c*) or *gcl*(*E*.*c*)/*garR*(*E*.*c*) did not display similar positive effect (Supplementary Fig. 5e). It suggested that Gcl/Hyi might be more effective in glyoxylate assimilation in *E*. *coli*. The negative results of Gcl/GlxR and Gcl/GarR were not caused by expressional problems since the Gcl/GlxR and Gcl/GarR combinations exhibited even higher enzymatic activities than Gcl/Hyi using crude extract assays after IPTG (isopropyl β-d-1-thiogalactopyranoside) pre-induction (Supplementary Fig. 6a and Supplementary Table 5). According to the above results, it showed Mtk/Mcl were the only heterologous enzymes required to achieve the complete pathway activity in *E*. *coli*.

### Effectiveness of the MCG pathway in *E*. *coli*

**Fig. 3:**
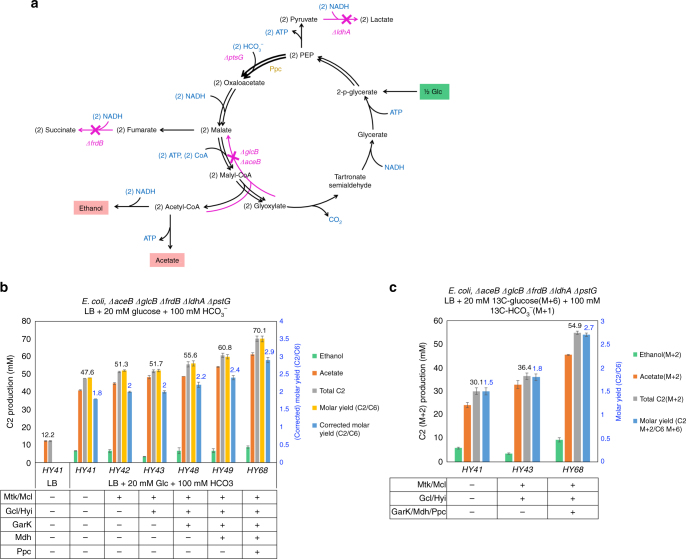
Functional demonstration of the MCG pathway in *E. coli*. **a** Rationale of gene deletions in the *E. coli* strain (*∆aceB ∆glcB ∆frdB ∆ldhA ∆ptsG*) used to investigate the effect of the MCG pathway. Deletions of *frdB* and *ldhA* were to eliminate the production of succinate and lactate. Deletions of *glcB* and *aceB* were to avoid the reverse reaction of Mtk/Mcl. Deletion of *ptsG* was to increase the intracellular PEP level. **b** Construction of the MCG pathway in *E. coli* increased the production of C2 compounds. After 24 h, 20 mM glucose was consumed. Expression of the complete pathway genes increased the titer of C2 compounds to 70.1 mM, which corresponds to the corrected C2/Glucose molar yield of 2.9, approaching the maximum theoretical value of 3 in *E. coli*. Molar yield (C2/C6) is calculated as the total C2 compounds (mM) produced divided by 20 mM glucose consumed. Corrected molar yield (C2/C6) refers to the total C2 compounds (mM) minus the C2 (12.2 mM acetate) produced in LB only, and then divided by 20 mM glucose consumed. **c** 13C isotopic measurement of total C2 (M + 2) production in LB medium supplemented with 20 mM 13 C uniformly labeled glucose (M + 6) and 100 mM 13C NaHCO_3_ (M + 1) under oxygen-limited condition. After 24 h, 20 mM glucose (M + 6) was consumed. Expression of the complete pathway genes achieved the C2(M + 2)/C6(M + 6) molar yield to 2.75, significantly exceeding the theoretical yield (=2) of wild type. Error bars are s.d., *n* = 3

To demonstrate the effectiveness of the whole pathway, an *E. coli* strain, *∆aceB ∆glcB ∆frdB ∆ldhA ∆pstG*, was created (Fig. 3a). LdhA^23^ and FrdABCD^24^ are lactate dehydrogenase and fumarate reductase which produce d-lactate and succinate, respectively. Their knockouts reduce carbon loss to these products, and channel the metabolic flux towards acetyl-CoA derived C2 compounds, acetate and ethanol, as the main fermentation products^25^. AceB^26^ and GlcB^27^ were deleted because they function as malate synthases that catalyze the reverse reaction of Mtk/Mcl. PtsG^28^ belongs to the PEP-dependent phosphotransferase system, and mediates uptake and phosphorylation of glucose. Its deletion increases the intracellular PEP pool^29^ and benefits the carbon flux towards to the OAA-forming direction through Ppc.

Enzymes of the MCG pathway were introduced into the strain *∆aceB ∆glcB ∆frdB ∆ldhA ∆pstG*. The cells were grown in Lysogeny Broth (LB) supplemented with 20 mM glucose and 100 mM bicarbonate under oxygen-limited condition. Glucose consumption and C2 compounds production, including acetate and ethanol, were measured after 24 h. The results showed that expression of *mtk*(*M*.*c*)/*mcl*(*M*.*e*) alone was only able to increase the titer of C2 compounds slightly compared to the control containing empty plasmid (Fig. 3b). Overexpression of *gcl*(*E*.*c*), *hyi*(*E.c*), *garK*(*E*.*c*), and *mdh*(*E*.*c*) further increased the C2 compound production. Additional expression of *ppc* from *Corynebacterium glutamicum* improved the titer of C2 compounds to 70.1 mM. After subtracting the C2 (12.2 mM acetate) produced in LB medium without glucose, the final corrected C2/C6 molar yield achieved 2.9 (Fig. 3b), approaching the maximum theoretical value of 3 in *E*. *coli* (Supplementary Fig. 2b**)**. Ppc(*C*.*g*) was used since it displayed much higher carboxylase activity with or without acetyl-CoA compared to the one from *E*. *coli* (Supplementary Fig. 6b and Supplementary Table 6).

To determine accurately the carbon fixation ability of the pathway, we grew the strain in LB medium supplemented with uniformly 13C-labeled glucose (M + 6) and 13C-bicarbonate (M + 1), and measured the production of double labeled C2 compounds (M + 2). The M + 2 form of the C2 produced and the M + 6 form of glucose consumed could evaluate the effect of the MCG pathway. If the MCG pathway is functioning, the (M + 2) C2/(M + 6) C6 molar yield should exceed 2, which is the maximum C2 carbon yield through the native glycolytic pathway. The results showed that the introduction of partial MCG pathway enzymes Mtk(*M*.*c*)/Mcl(*M*.*e*)/Gcl(*E*.*c*)/Hyi(*E*.*c*) produced 36.4 mM isotope-labeled C2 compounds (M + 2), achieving the C2/C6 molar yield of 1.82 compared to 1.51 of the control (Fig. 3c). Additional expression of remaining MCG pathway genes, including *garK*(*E*.*c*), *mdh*(*E*.*c*), and *ppc*(*C*.*g*), increased the total C2 compounds (M + 2) to 54.9 mM (Fig. 3c), which raised the C2/C6 molar yield to 2.75, significantly exceeding the theoretical yield (=2) of wild type. These results conclusively demonstrated that the MCG pathway was able to achieve efficient acetyl-CoA synthesis through carbon fixation in *E*. *coli*.

### MCG pathway increased acetyl-CoA pool in cyanobacteria

To investigate the effect of MCG coupling with the CBB cycle, we constructed the MCG pathway in cyanobacteria *S*. *elongatus PCC7942*. The genes, *ppc*(*E*.*c*), *mdh*(*E*.*c*), *mtkAB*(*M*.*c*), and *mcl*(*M*.*e*), were integrated into neutral site I^30^ of the genome, and the remaining genes, *gcl*(*Cupriavidus necator*), *glxR*(*E*.*c*), and *garK*(*E*.*c*), were integrated into neutral site II^30^. Gcl(*C*.*n*) displayed higher glyoxylate-condensation activity than the one from *E*. *coli* in cyanobacteria (Supplementary Fig. 7a). After verification of chromosomal integration by colony PCR and enzyme assays, the resulting cyanobacterial strains (McG-140, McG-142, and McG-145) were grown under 50 μE/s/m^2^ continuous light, and cell growth was measured. Unlike the NOG pathway, introduction of the complete MCG pathway enzymes in the strain McG-140 did not negatively affect growth compared to wild type (Supplementary Fig. 7b). Expression of the complete pathway genes also improved cell growth compared with the controls (McG-142 and McG-145) that expressed partial pathway genes.

**Fig. 4:**
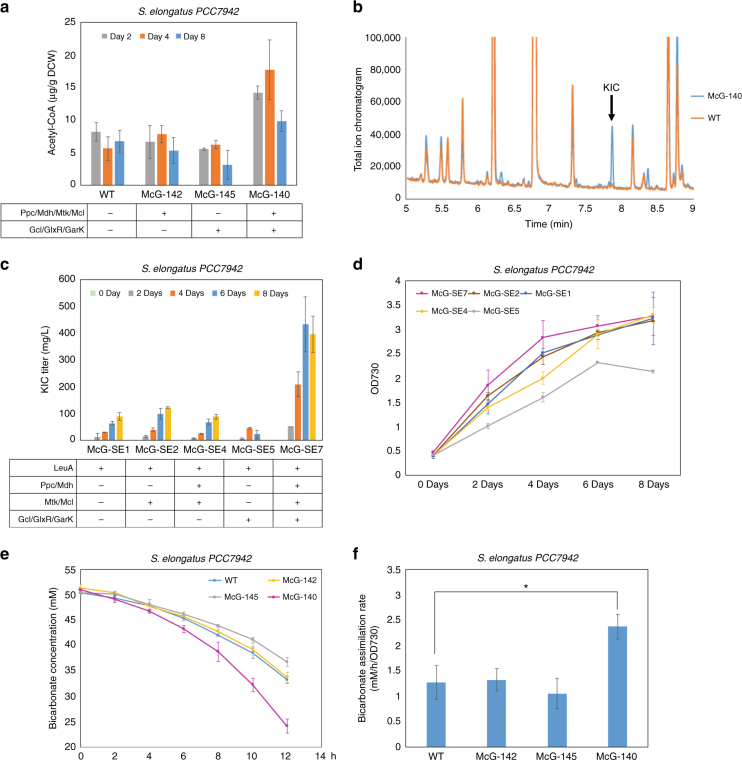
MCG pathway increased the intracellular acetyl-CoA pool and enhanced carbon fixation in *S. elongatus*. **a** Expression of the complete pathway genes in the strain McG-140 increased the intracellular acetyl-CoA level compared to wild type and the controls expressing partial pathway genes. Error bars are s.d., *n* = 3. **b** GC-MS identification of ketoisocaproate (KIC) production in the McG-140 culture. **c** The strain McG-SE7 significantly increased the KIC production compared to the controls with expression of the partial pathway genes. The KIC titer in the McG-SE7 achieved the highest amount of 433 mg/L. Error bars are s.d., *n* = 3. **d** The strain McG-SE7 promoted cell growth to saturation faster than all the controls. Strain designations are defined as in **c**. Error bars are s.d., *n* = 3. **e**, **f** The McG-140 strain assimilated more bicarbonate than wild type and the controls expressing the partial pathway (**e**), and increased the bicarbonate assimilation rate (**f**) as well. The bicarbonate assimilation rate (**f**) was shown between the eighth to tenth hour of **e**, which displayed the most obvious differences. Strain designations are defined as in **a**. Error bars are s.d., *n* = 3. *P < 0.05 (*t*-test, two tails)

To evaluate the effect of the MCG pathway, intracellular acetyl-CoA level was determined. The strain (McG-140) expressing the complete pathway genes markedly increased the acetyl-CoA level compared to wild type and the controls (Fig. 4a). Supernatant of cyanobacterial cultures was analyzed by high-performance liquid chromatography (HPLC). The wild-type strain does not produce any organic compounds detectable on HPLC. We hypothesized that the increased acetyl-CoA level in the strain McG-140 would be converted to acetate. However, no acetate was detected in the strains expressing the pathway genes as well as in the wide type. Instead, we discovered two unknown peaks on the chromatogram with retention times at 17 and 20 min (Supplementary Fig. 7c and d), specifically appeared in the stain McG-140. The first unknown peak gradually disappeared after a few days, while the second peak accumulated by days. The second unknown peak matched the retention time of ketoisocaproate (KIC) and the production of this compound was further confirmed by gas chromatograph-mass spectrometry (GC-MS) (Fig. 4b and Supplementary Fig. 7e). Previously we demonstrated the production of isobutanol in *S*. *elongatus* with expression of only *kivd* and *yqhD*^31,30^, which indicated an abundant intracellular pool of ketoisovalerate (KIV) in this organism. In this case, KIV was converted to KIC by condensation with acetyl-CoA through the leucine biosynthesis pathway (Supplementary Fig. 7g). Therefore, the production of KIC was consistent with the increased production of acetyl-CoA by expression of the MCG genes.

To evaluate the effectiveness of the MCG pathway accurately, we integrated *leuA*(*E*. *coli*) into neutral site III^32^ of the cyanobacterial strains (McG-140, McG-142, and McG-145), resulting in McG-SE7, McG-SE4, and McG-SE5. LeuA(*E*.*c*)^33^ functions as 2-isopropylmalate synthase that catalyzes the incorporation of acetyl-CoA to KIV for KIC synthesis (Supplementary Fig. 7g). Only the strain McG-SE7, expressing the complete pathway genes, significantly increased the KIC production compared to the controls (McG-SE4 and McG-SE5) expressing different groups of the pathway genes (Fig. 4c), suggesting that the MCG pathway was effective. The KIC titer in the McG-SE7 reached the highest amount of 433 mg/L on the sixth day. Although overexpression of *leuA*(*E*.*c*) caused growth retardation (Supplementary Fig. 7f), the McG-SE7 strain displayed a similar growth pattern as McG-140 in that it grew faster to saturation compared to various control strains that expressed partial pathway genes (Fig. 4d). McG-SE7 also showed the improved cell growth than the McG-SE1 strain with *leuA*(*E*.*c*) expression alone.

### MCG pathway increased carbon fixation in cyanobacteria

To investigate the effect of expressing the MCG pathway on carbon fixation, bicarbonate assimilation was determined in cyanobacterial cultures by using nuclear magnetic resonance (NMR) spectroscopy^34^ (Supplementary Fig. 8a and 8b). Each cyanobacterial strain was grown for 4 days, and normalized to culture OD730 about 1 in fresh BG-11^30^ medium with 50 mM 13C-labeled bicarbonate. Bicarbonate concentration in the medium was then measured at subsequent time points. The results showed that the McG-140 strain with IPTG induction assimilated more bicarbonate than wild type and the controls (McG-142 and McG-145) expressing partial pathway genes (Fig. 4e). About 25.7 mM 13C-HCO_3_^−^ were consumed by the McG-140 strain compared to 16.7 mM of wild type after 12 h incubation. The McG-140 strain increased the specific bicarbonate assimilation rate, while McG-142, which expressed only *ppc*(*E.c*)/*mdh*(*E*.*c*)/*mtkAB*(*M*.*c*)/*mcl*(*M*.*e*), or McG-145, which expressed only *gcl*(*C*.*n*)/*glxR*(*E*.*c*)/*garK*(*E*.*c*), did not show any effect (Fig. 4f, Supplementary Table 7 and Supplementary Table 8). This result indicated that the increased bicarbonate consumption could not be attributed to the increased Ppc activity alone. Photosynthetic O_2_ production was determined under the same conditions except using unlabeled bicarbonate. The O_2_ evolution of McG-140 was similar to that of wild type under 50 μE/s/m^2^ light condition (Supplementary Fig. 8d), suggesting that expression of the pathway genes did not affect the ATP production rate by photosystems^30,35^. Thus, it appeared that coupling the MCG pathway with the CBB cycle increased carbon fixation possibly through more efficient utilization of photosystem-generated energy in cyanobacteria, as predicted in Table 1.

## Discussion

Previous work to improve the CBB cycle mainly focused on engineering the cycle enzymes^36–39^. Here we sought to augment the CBB cycle by constructing a synthetic pathway to complement the deficiency of the CBB cycle. The MCG pathway, coupling with the CBB cycle, allows photosynthetic cells to utilize only 5.5 ATP and 1.5 Rubisco turnovers to produce one acetyl-CoA from CO_2_ equivalents (Table 1), as opposed to the native pathway that requires seven ATP and three Rubisco turnovers. The MCG pathway has no oxygen sensitivity issue, and does not compete with the major existing metabolic pathways. More importantly, the MCG pathway provides an additional route for CO_2_ fixation via Ppc, one of the most robust and active carbon fixing enzymes. The reduced dependence on Rubisco and use of an additional CO_2_ fixing enzyme increased carbon fixation. Although the thaumarchaeal HP/HB cycle^40^, found in *Nitrosopumilus maritimus*, can synthesize one acetyl-CoA from CO_2_ equivalents with the expense of only four ATP (Supplementary Table 1), it may be more challenging to implement this cycle in photosynthetic organisms. Since the thaumarchaeal HP/HB cycle requires 16 enzymes to achieve the complete cycle and some of the enzymes have not been characterized yet^40^.

In addition to carbon fixation, the MCG pathway also can reduce carbon loss in photorespiration by converting glycolate to acetyl-CoA without net carbon loss (Fig. 1b). A previous strategy utilizes a bacterial glycolate assimilation route^41^ to save carbon loss from the endogenous photorespiration pathway. However, it still assimilates two molecules of glycolate to produce only one acetyl-CoA. The theoretical carbon yield is 50% (Table 2). The MCG pathway, coupling with glycolate dehydrogenase, can convert each glycolate to stoichiometric amount of acetyl-CoA with 100% carbon yield. Thus, coupling the MCG pathway with the CBB cycle in photosynthetic organisms may be a practical approach to improve photosynthetic carbon fixation.

## Methods

### Protein synthesis and purification

Ppc, Eno, and Mdh were purchased from Sigma-Aldrich. Mtk(*M*.*c*) has two subunits, and the gene coding for each subunit was fused with a 6xHis-tag at the C-terminal and cloned into the same operon. The remaining genes, *mcl*(*M*.*e*), *gcl*(*E*.*c*), *glxR*(*E*.*c*), *gark*(*E*.*c*), and *pps*(*E*.*c*), were fused with a His-tag at the N-terminal. All genes were cloned under the T7 promoter and transformed into *E*. *coli BL21* (*DE3*) for expression. Overnight culture was inoculated (2% vol/vol) into fresh LB medium. Cells were grown at 37 °C with agitation at 250 rpm to mid-log phase (OD600 of 0.4–0.6), and induced for gene expression by 0.1 mM IPTG (Zymo Research) for additional 6 h at 30 °C. Cell pellets were lysed with 0.1 mm diameter glass beads at 4 °C. Proteins were purified by His-Spin protein mini-prep columns (Zymo Research). Concentrations of purified proteins were measured using BioRad protein assay kit, and the purity was verified by sodium dodecyl sulfate polyacrylamide gel electrophoresis with coomassie staining.

### Demonstration of the MCG pathway in vitro

Pyruvate as the initial substrate: The assay was set up at 37 °C in a final volume of 400 μL containing 50 mM Tris-Cl (pH 7.5), 5 mM MgCl_2_, 0.5 mM TPP, 2 mM pyruvate, 6 mM NaHCO_3_, 8 mM CoA, 10 mM ATP, 10 mM NADH with enzymes including MtkAB, Mcl, Gcl, GlxR, GarK, Ppc, Eno, Mdh, and Pps.

Glyoxylate as the initial substrate: The assay was set up at 37 °C in a final volume of 400 μL containing 50 mM Tris-Cl (pH 7.5), 5 mM MgCl_2_, 0.5 mM TPP, 2 mM glyoxylate, 6 mM NaHCO_3_, 4 mM CoA, 6 mM ATP, 6 mM NADH with enzymes including MtkAB, Mcl, Gcl, GlxR, GarK, Ppc, Eno, and Mdh.

Fifty microliters of the reaction mixture was incubated with 10% formic acid to stop reactions. The detection method of glyoxylate or pyruvate was optimized according to the protocol^42^. Briefly, after incubation with formic acid, samples were reacted with 2 mM phenylhydazine to form glyoxylate(pyruvate)-phenylhydrazone, which displayed absorbance at 324 nm and could be separated with NADH peak by HPLC (Agilent 1200) with a C18 column (Thermo Fisher Scientific). The acetyl-CoA amount could be quantified by the C18 column through photodiode array detector at 260 nm. Protein amount used for each assay was described in Supplementary Methods.

### Construction of *E*. *coli* strains

All *E*. *coli* strains used in this study are listed in Supplementary Table 10. *JCL16* was used to create the acetyl-CoA auxotroph *∆aceEF ∆poxB ∆pflB*. *MC4100* was used to construct the pyruvate auxotroph *∆maeAB ∆pckB*. The remaining *E*. *coli* strains used *BW25113* as the parental strain for construction. Gene deletion was performed by P1 transduction with single knockout strain from the Keio collection.

### Plasmid construction

Plasmids used in the study are listed in Supplementary Table 10. All plasmids were constructed by using Gibson DNA assembly^43^. The primers used for the cloning are shown in Supplementary Table 11^44^.

### Growth rescue of *E*. *coli* strains

Overnight *E*. *coli* culture was inoculated (2% vol/vol) into fresh LB medium. *E*. *coli* culture was allowed to grow at 37 °C in a rotary shaker (250 rpm) to an OD600 of 0.4–0.6. About 0.1 mM IPTG was added to induce protein synthesis at 30 °C for 6 h. One milliliter of culture was harvested and washed three times with equal volume of minimal medium. Sixty microliters of culture was inoculated (2% vol/vol) into 3 mL minimal medium for growth testing at 37 °C. Minimal medium contains M9 salts (12.8 g/L Na_2_HPO_4_**·**7H_2_O, 3 g/L KH_2_PO_4_, 0.5 g/L NaCl, 1 g/L NH_4_Cl), 1 mM MgSO_4_, 0.1 mM CaCl_2_, 0.1 mg/mL thiamine hydrochloride, 0.1 mM IPTG, appropriate antibiotics (kanamycin 40 μg/mL, ampicillin 100 μg/mL, or spectinomycin 50 μg/mL) and carbon sources (all from Sigma-Aldrich) as noted in the study. The growth experiments were performed aerobically.

### Measurement of C2 compounds in *E. coli* culture

Overnight *E*. *coli* culture was inoculated (2% vol/vol) into fresh 20 mL LB medium. *E*. *coli* culture was grown at 37 °C in a rotary shaker (250 rpm) to an OD600 of 0.4–0.6. About 0.2 mM IPTG was used to induce gene expression at 30 °C for 6 h. Six milliliters of culture was harvested and resuspended into 2 mL fresh LB medium supplemented with 20 mM glucose, 100 mM bicarbonate, and 0.1 mM IPTG with appropriate antibiotics. Two milliliters culture (OD600 about 10) was fermented in a BD vacutainer glass tube capped at 37 °C for 24 h. For isotope labeling experiments, *E*. *coli* culture was prepared as stated above except using d-Glucose-13C6 (from Santa Cruz Biotechnology, Dallas, TX.) and sodium bicarbonate-13C (from Sigma-Aldrich). To measure C2 compounds, culture was centrifuged at 15,000 *g* for 5 min, and supernatant was diluted for five times and filtered by Amicon 10 kDa protein filters (EMD-Amicon). Twenty microliters of sample was applied to the Agilent 1200 HPLC system with a Bio-Rad Aminex HPX87 column (30 mM H_2_SO_4_; 0.4 mL/min; column temperature, 30 °C). Acetate was detected by photodiode array detector at 210 nm. Glucose consumption was quantified by a biochemistry analyzer 2300 (YSI). Ethanol was measured by a GC-flame ionization detector (FID) (Agilent Technologies). 1-Propanol was used as the internal standard. 13C-labeled acetate (M + 2) and ethanol (M + 2) were determined by GC-MS (Agilent Technologies) as described in Bogorad et al. (2014)^45^.

### Construction of cyanobacterial strains

*S*. *elongatus* culture was grown to mid-log phase (OD730 of 0.4–0.6) and incubated with 2 μg of plasmid DNA overnight in the dark. *S*. *elongates* culture was then spread on modified BG-11^30^ plates supplemented with appropriate antibiotics for selection of successful recombination. Spectinomycin (20 μg/mL), 10 μg/mL kanamycin, and 10 μg/mL gentamicin were used in BG-11 agar plates as needed. Strain segregation was confirmed by colony PCR. Modified BG-11 contains 1.5 g/L NaNO_3_, 0.0272 g/L CaCl_2_·2H_2_O, 0.012 g/L ferric ammonium citrate, 0.001 g/L EDTA disodium, 0.04 g/L K_2_HPO_4_, 0.0361 g/L MgSO_4_·7H_2_O, 0.02 g/L Na_2_CO_3_, 1× trace minerals, and 0.0088 g/L sodium citrate. 1000× trace minerals includes 2.86 g/L H_3_BO_3_, 1.81 g/L MnCl_2_·4H_2_O, 0.222 g/L ZnSO_4_·7H_2_O, 0.39 g/L Na_2_MoO_4_·2H_2_O, 0.079 g/L CuSO_4_·5H_2_O, 0.049 g/L Co(NO_3_)_2_·6H_2_O.

### Growth measurement of cyanobacterial strains

Seed culture was grown in 20 mL of BG-11 with 50 mM NaHCO_3_ and appropriate antibiotics. The strains were grown under 50 µE/s/m^2^ light condition with continuous shaking at 30 °C. The cyanobacterial culture was fed with 50 mM NaHCO_3_ (add 1 mL of 1 M NaHCO_3_ dissolved in BG-11) every day until OD730 reached 2–3. Then the culture was diluted to OD730 of 0.5, and grown in 5 mL of BG-11 medium with 50 mM NaHCO_3_, appropriate antibiotics, 40 µM d-pantothenic acid (hemicalcium salt), 0.2 mM thiamine pyrophosphate, and 0.5 mM IPTG. The culture was grown under 50 µE/s/m^2^ light intensity in a BD vacutainer glass tube at 30 °C. A low oxygen condition was created by flushing the tube headspace with nitrogen once per day in order to decrease acetyl-CoA consumption by endogenous TCA cycle. The cyanobacterial culture was fed everyday with 50 mM bicarbonate (add 250 μL of 1 M NaHCO_3_ dissolved in BG-11). The growth was monitored by a Beckman Coulter DU800 spectrophotometer at 730 nm.

### Measurement intracellular acetyl-CoA level in cyanobacteria

For measurement of intracellular acetyl-CoA, cyanobacterial culture was prepared as above, and pellet was lysed with 0.1 mm diameter glass beads at 4 °C. The intracellular acetyl-CoA level was determined by Acetyl-Coenzyme A Assay Kit (from Sigma-Aldrich).

### Measurement of bicarbonate consumption in cyanobacteria

For measurement of bicarbonate consumption, the cyanobacterial culture (OD730 about 3) was spin down and normalized to OD about 1 by fresh BG-11 medium with 50 mM 13C-bicarbonate. Five milliliters of culture was grown under a low oxygen condition in a sealed tube at 50 μE/s/m^2^ light intensity. OD was monitored and 0.5 mL of culture was used for measurement of bicarbonate concentration. The culture was centrifuged at 12,000 *g* for 5 min and supernatant was diluted for four times. Nine hundred and fifty microliters of the sample, mixed with 50 μL D_2_O (from Sigma-Aldrich), was used for NMR spectroscopy. The bicarbonate fixation rate was calculated as: bicarbonate consumption (mM)/time interval (2 h)/average OD730.

### Measurement oxygen production in cyanobacteria

For measurement of O_2_ production, the cyanobacterial culture was prepared under the same conditions as the measurement of bicarbonate consumption except using 50 mM unlabeled bicarbonate^46^. Oxygen production was measured by the Oxygraph System (from Hansatech Instruments)^47^.

### Ketoisocaproate production in cyanobacteria culture

Fifty microliters of supernatant of cyanobacteria culture was mixed with 1.8 mL of solvent (MeOH:CHCl_3_:H_2_O as 5:3:2 vol/vol) containing 20 mg/L xylitol, and then incubated at −20 °C for 1 h. The sample was centrifuged. Three hundred microliters of supernatant were freeze-dried by vacuum centrifugation. Derivatization of GC samples: 50 μL of methoxyamine hydrochloride with 20 mg/mL pyridine was added to the freeze-dried sample, and incubated at 30 °C for 90 min with 1200 rpm shaking. Twenty-five microliters of *N*-methyl-*N*-(trimethylsilyl) trifluoroacetamide (MSTFA) was added and incubated at 37 °C for 30 min with shaking at 1200 rpm. The samples were analyzed within 24 h by GC-MS.‬‬‬‬‬‬‬‬‬‬‬‬‬‬‬‬‬‬‬‬‬‬‬‬‬‬‬‬‬‬‬‬‬‬‬‬‬‬‬‬‬‬‬‬‬‬‬‬‬‬‬‬‬‬‬‬‬‬‬‬‬‬‬‬‬‬‬‬‬‬‬‬‬‬‬‬‬‬‬‬‬‬‬‬‬‬‬‬‬‬‬‬‬‬‬‬‬‬‬‬‬‬‬‬‬‬‬‬‬‬‬‬‬‬‬‬‬‬‬‬‬‬‬‬‬‬‬‬‬‬‬ For measurement of ketoisocaproate production, the cyanobacterial culture was centrifuged at 15,000 *g* for 5 min. Supernatant was analyzed by the Agilent 1200 HPLC system equipped with a BioRad HPX87 column (30 mM H_2_SO_4_; 0.6 mL/min; column temperature, 30 °C). Ketoisocaproate concentration was monitored by a photodiode array detector at 210 nm.

### Data analysis

Data are presented as mean ± s.d. (standard deviation) unless otherwise indicated in figure legends. For strain growth and production assays, three biological replicates of each strain were measured.

### Data availability

All the genes used this study are listed in Supplementary Table 9. Their sequences can be obtained by searching accession ID and the associated organism in Biocyc (https://biocyc.org/). All other relevant data are available from the authors upon request.

## Electronic supplementary material

Supplementary Information
