# Supplementary material for: Augmenting the Calvin–Benson–Bassham cycle by a synthetic malyl-CoA-glycerate carbon fixation pathway
Source: Nat Commun. 2018 May 22;9:2008. doi: 10.1038/s41467-018-04417-z (PMC5964204; doi:10.1038/s41467-018-04417-z)
Supplement: Supplementary file 1 — Supplementary Information [file 41467_2018_4417_MOESM1_ESM.docx]

**Augmenting the Calvin-Benson-Bassham cycle by a synthetic malyl-CoA-glycerate carbon fixation pathway**

Yu *et al.*

**Supplementary Note 1**

Demonstration of the feasibility of the MCG pathway by *in vitro* production of acetyl-CoA

We set up an *in vitro* system to demonstrate the feasibility of the MCG pathway using purified enzymes. We cloned *mtk* from *M. capsulatus*, *mcl* from *M. extorquens*, and *gcl* (glyoxylate carboligase), *glxR* (tartronate semialdehyde reductase), *garK* (glycerate kinase) and *pps* (PEP synthase) from *E. coli* with a 6xHis-tag for one-step protein purification (Supplementary Fig. 4g). Other enzymes, namely Ppc, enolase (Eno) and malate dehydrogenase (Mdh), were acquired from Sigma. The activity of each purified enzyme was measured by *in vitro* assays (Supplementary Fig. 4a-4d and Supplementary Table 4). We then set up two multi-enzyme cascade reactions, which together could form the complete MCG pathway, to test their functions. The results showed both sub-pathways worked as expected *in vitro* (Supplementary Fig. 4e and 4f).

To demonstrate the whole pathway *in vitro*, 2 mM pyruvate or glyoxylate was added to the mixtures containing all the enzymes shown in Figure 1a (with Pps) and Figure 1b, respectively, with supplementation of cofactors. 50 units of each enzyme was used, and the production of acetyl-CoA was measured in a short period of time in order to avoid the decomposition of acetyl-CoA and thermal instability of purified enzymes.

**Supplementary Methods**

*In vitro* enzyme assays

Mtk/Mcl assay: Mtk performs the ATP-dependent condensation of malate and CoA to form malyl-CoA, and Mcl cleaves malyl-CoA into acetyl-CoA and glyoxylate. The latter reacts with phenylhydazine to form glyoxylate-phenylhydrazone that displays absorbance at 324 nm (nanometer). The assay was set up at 37°C in a final volume of 200 μL containing 50 mM Tris-Cl (pH 7.5), 5 mM MgCl_2_, 2 mM phenylhydrazine, 10 mM malate, 2.5 mM ATP, 2 mM CoA, 15 ug MtkAB and 5 ug Mcl purified protein.

Gcl/GlxR assay: Gcl catalyzes the reaction of condensing two glyoxylate to produce one tartronate semialdehyde, GlxR reduces tartronate semialdehyde to glycerate with oxidation of NADH, which can be recorded at 340 nm. The assay was set up at 37°C with a final volume of 200 μL containing 50 mM Tris-Cl (pH 7.5), 5 mM MgCl_2_, 0.5 mM TPP (thiamine pyrophosphate), 5 mM glyoxylate, 0.25 mM NADH, 10 ug Gcl and 5 ug GlxR purified protein.

GarK assay: GarK catalyzes the ATP-dependent phosphorylation of glycerate to produce 2-phosphoglycerate and ADP. Pyk/Ldh (pyruvate kinase/lactate dehydrogenase) enzyme mixture (from Sigma-Aldrich) was used to measure ADP formation in the reaction. Pyk uses ADP as cofactor to catalyze the reaction of converting PEP to pyruvate, and Ldh reduces pyruvate to lactate by oxidation of NADH, which is recorded at 340 nm. The assay was set up at 37°C in a final volume of 200 μL containing 50 mM Tris-Cl (pH 7.5), 5 mM MgCl_2_, 5 mM glycerate, 2 mM PEP, 2.5 mM ATP, 0.25 mM NADH, 10 ug GarK purified protein and 1 ul of Pyk/Ldh mixture.

Pps/Ppc/Mdh: Pps can phosphorylate pyruvate to PEP, Ppc catalyzes the carboxylation reaction of PEP to generate oxaloacetate, and Mdh reduces oxaloacetate to form malate with oxidation of NADH, which can be recorded at 340 nm. The assay was set up at 37°C in a final volume of 200 μL containing 50 mM Tris-Cl (pH 7.5), 5 mM MgCl_2_, 2 mM pyruvate, 5 mM NaHCO_3_, 2.5 mM ATP, 0.25 mM NADH, 5 ug Pps, 0.5 ul of Ppc, and 0.5 ul of Mdh.

Mtk/Mcl/Gcl/GlxR assay: Mtk/Mcl split malate to produce acetyl-CoA and glyoxylate. The latter can be condensed to form tartronate semialdehyde and further reduced to glycerate, catalyzed by Gcl/GlxR. NADH oxidation is used to record the reaction. The assay was set up at 37°C in a final volume of 200 μL containing 50 mM Tris-Cl (pH 7.5), 5 mM MgCl_2_, 0.5 mM TPP, 2.5 mM ATP, 2 mM CoA, 0.25 mM NADH, 10 mM malate, 20 ug MtkAB, 5 ug Mcl, 10 ug Gcl and 5 ug GlxR.

GarK/Eno/Ppc/Mdh assay: GarK/Eno catalyze the reactions to convert glycerate to PEP, Ppc catalyzes the carboxylation reaction of PEP to form OAA, and Mdh can reduce OAA to generate malate. NADH oxidation can be recorded at 340 nm. The assay was set up at 37°C in a final volume of 200 μL containing 50 mM Tris-Cl (pH 7.5), 5 mM MgCl_2_, 2 mM glycerate, 5 mM NaHCO_3_, 2.5 mM ATP, 0.25 mM NADH, 10 ug Gark, 0.5 ul of Ppc, 0.5 ul of Eno and 0.5 ul of Mdh.

**
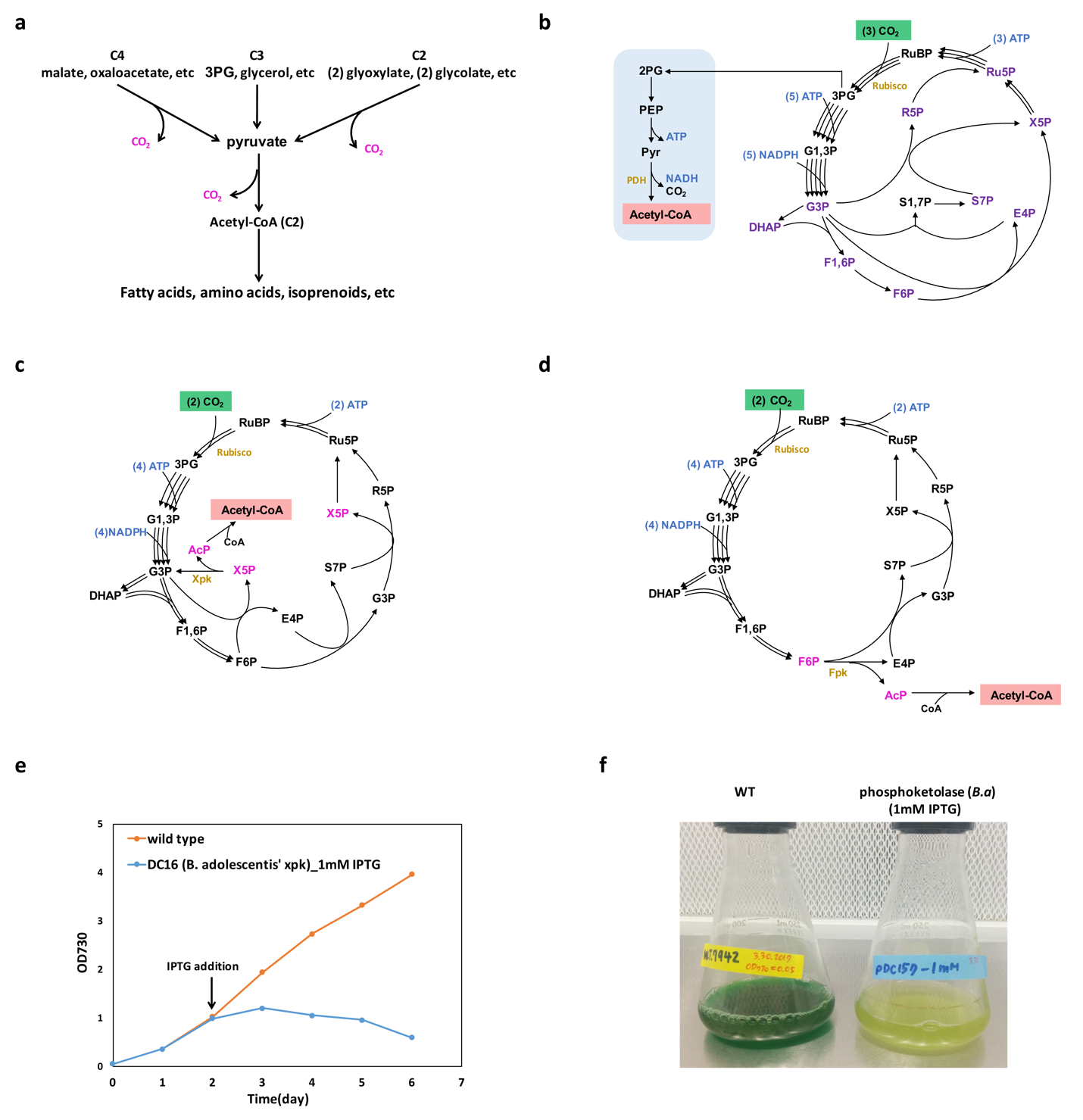
**

**Supplementary Figure 1. The incompatibility between the CBB cycle with the NOG pathway.**

(**a**) Acetyl-CoA synthesis, decarboxylation from C3 sugars, is a major carbon loss in the cell. Meanwhile, when C4 or C2 compounds are metabolized to acetyl-CoA, carbon loss is more serious. One molecule of C4 metabolite converts to one acetyl-CoA by losing two carbons. Two molecules of C2 metabolite, such as glyoxylate or glycolate, can generate only one acetyl-CoA.

(**b**) Illustration of the Calvin-Benson-Bassham (CBB) cycle. The CBB cycle is not evolved for optimal synthesis of acetyl-CoA. Blue frame indicates the acetyl-CoA synthesis from decarboxylation of the CBB cycle product, 3-phosphoglycerate. The CBB cycle and NOG pathway both compete for the same intermediates (labelled as purple). 3PG: 3-phosphoglycerate. G1,3P: 1,3-Bisphosphoglycerate. G3P: glyceraldehyde-3-phosphate. DHAP: dihydroxyacetone phosphate. F1,6P: fructose 1,6-bisphosphate. F6P: fructose-6-phosphate. E4P: erythrose-4-phosphate. S7P: sedoheptulose-7-phosphate. S1,7P: sedoheptulose-1,7-bisphosphate. R5P: ribose-5-phosphate. X5P: xylulose-5-Phosphate. Ru5P: ribulose-5-phosphate. RuBP: ribulose-1,5-bisphosphate. 2PG: 2-phosphoglycerate. Pyr: pyruvate.

(**c**-**d**) Illustration of integrating the NOG pathway with the CBB cycle. Phosphoketolase^8,12^ can irreversibly cleave the carbon-carbon bond of either xylulose 5-phosphate (Xu5P, optimal substrate) or fructose 6-phosphate (F6P). (**c**) shows the CBB cycle integrates with the NOG pathway (phosphoketolase acts as Xpk activity) and (**d**) shows the CBB cycle integrates with the NOG pathway (phosphoketolase acts as Fpk activity). Integration of the CBB cycle with the NOG pathway facilitates photosynthetic cells to use only 2 Rubisco turnovers and 6 ATP to synthesize one acetyl-CoA.

(**e**-**f**) Expression of *xpk*(*Bifidobacterium adolescentis*) seriously inhibited cyanobacterial growth. The picture (**f**) was taken at the 4th day after 1mM IPTG addition.


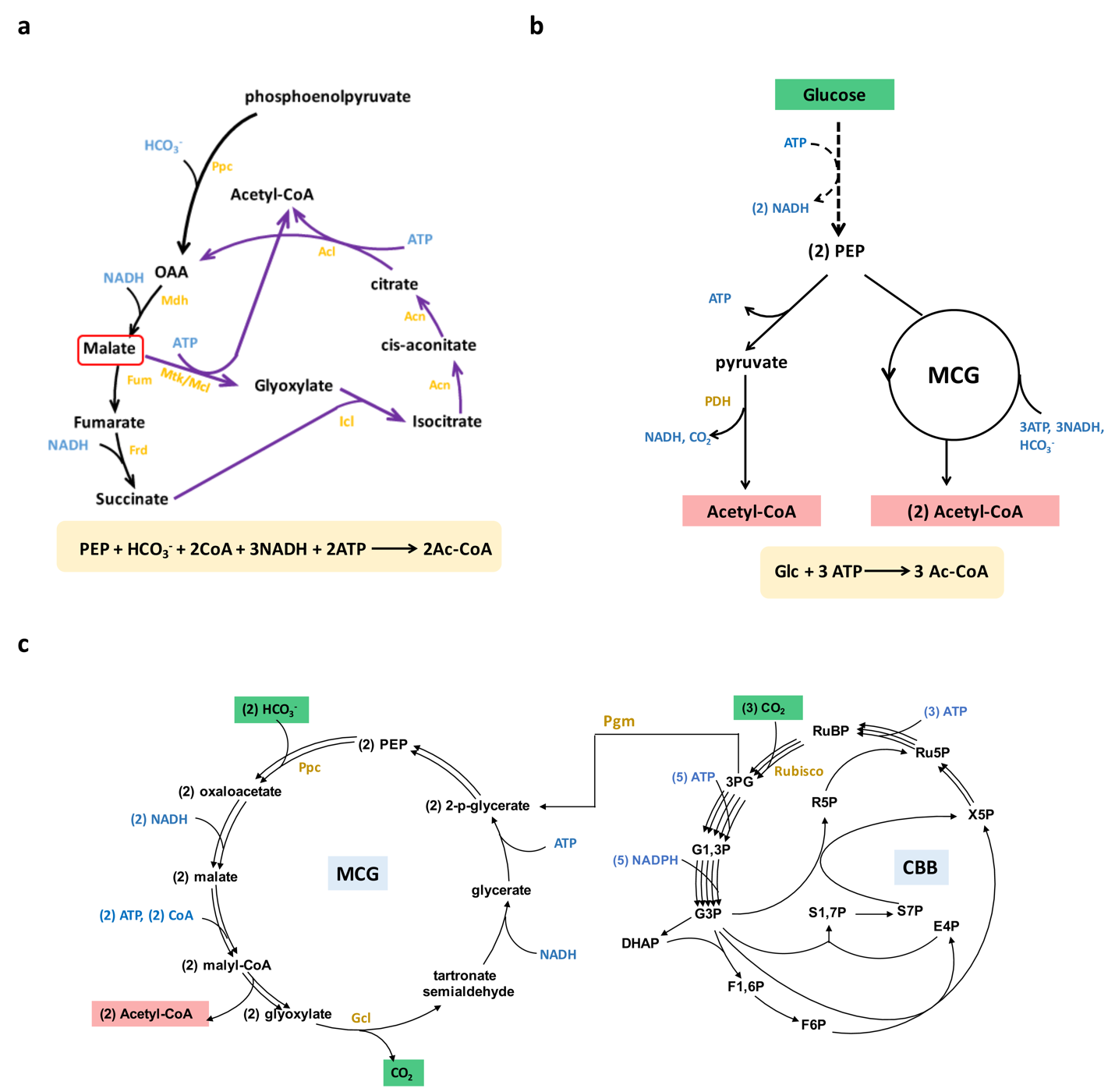


**Supplementary Figure 2. Design of the rGS-citrate and MCG pathway.**

(**a**) The rGS-citrate pathway allows one PEP to generate two molecules of acetyl-CoA by fixation of an additional CO_2_ equivalent. However, the rGS-citrate pathway is not robust, which might be caused by imbalanced carbon flux at the malate node (Red box) predicted by computational analysis^14^. The purple lines indicated the feasibility of the part of the pathway demonstrated in an oxaloacetate auxotrophic *E. coli*^13^. The net reaction is shown in the yellow box. FumC: fumarase C. Frd: fumarate reductase. Mtk: malate thiokinase. Mcl: malyl-CoA lyase. Icl: isocitrate lyase. Acn: aconitate hydratase. Acl: ATP-citrate lyase.

(**b**) In *E. coli*, the MCG pathway can convert one glucose to three acetyl-CoA, achieving complete carbon conservation. The net reaction is shown in the yellow box.

(**c**) The MCG pathway, coupling with the CBB cycle, can synthesize each acetyl-CoA from atmospheric CO_2_ with the expense of 5.5 ATP and 4 NADH. The cells can use phosphoglycerate mutase (Pgm) to convert 3-phosphoglycerate, the CBB cycle product, to 2-phosphoglycerate, then entering into the MCG pathway.

**
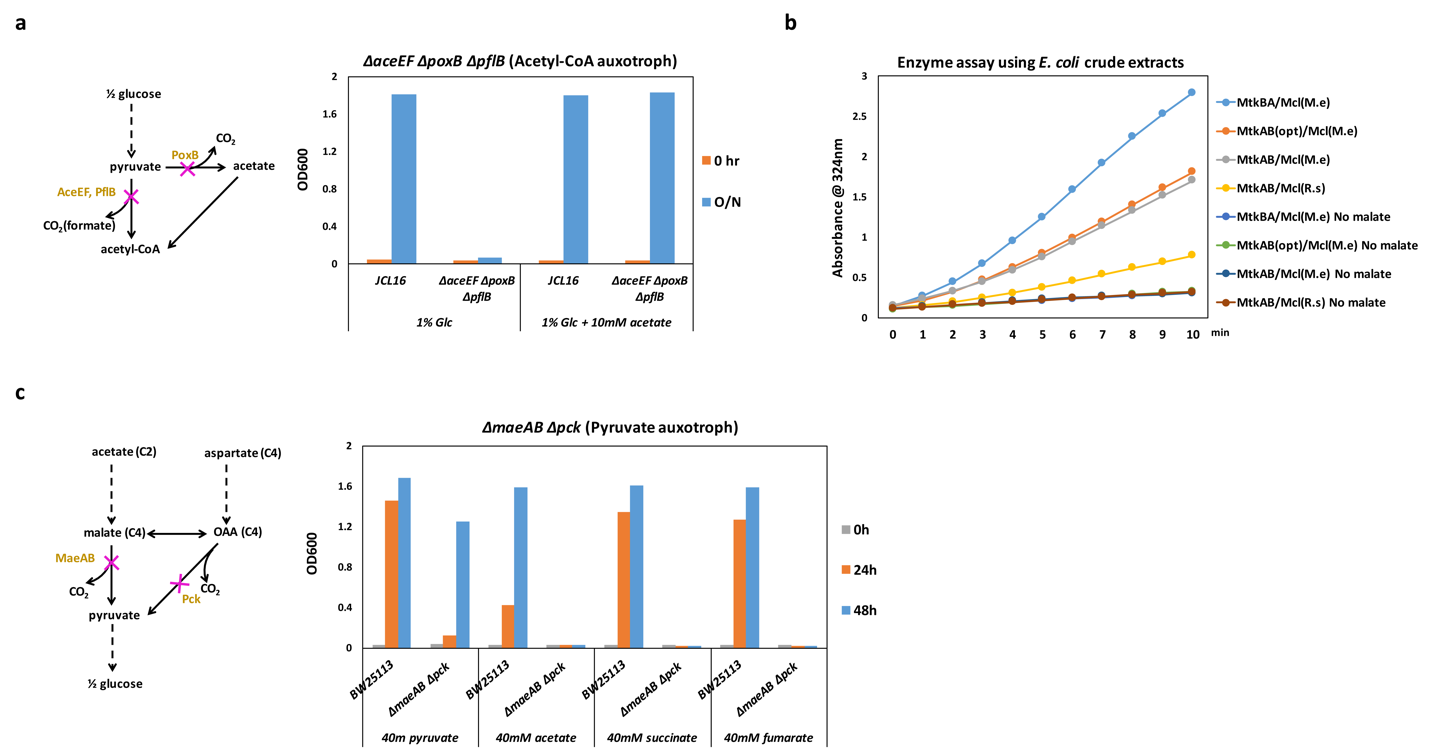
**

**Supplementary Figure 3. Introduction of *E. coli* auxotrophic strains used in this study.**

(**a**) Characterization of the *E. coli* strain *∆aceEF ∆poxB ∆pflB* as an acetyl-CoA auxotroph. The strain cannot grow in minimal medium with glucose as the sole carbon source unless supplemented with acetate. *JCL16* was used as the wild type control. 0 hr: Time Zero, O/N: overnight.

(**b**) The activity of Mtk/Mcl measured by crude extracts from Figure 2b. 10 ug (total protein amount) crude extracts were used for measurement.

(**c**) Characterization of the *E. coli* strain *∆maeAB ∆pck* as a pyruvate auxotroph. The strain cannot grow in minimal medium with C2 or C4 compound as the sole carbon source, but can grow on pyruvate (C3) or its upstream compounds. *BW25113* was used as the wide type control.


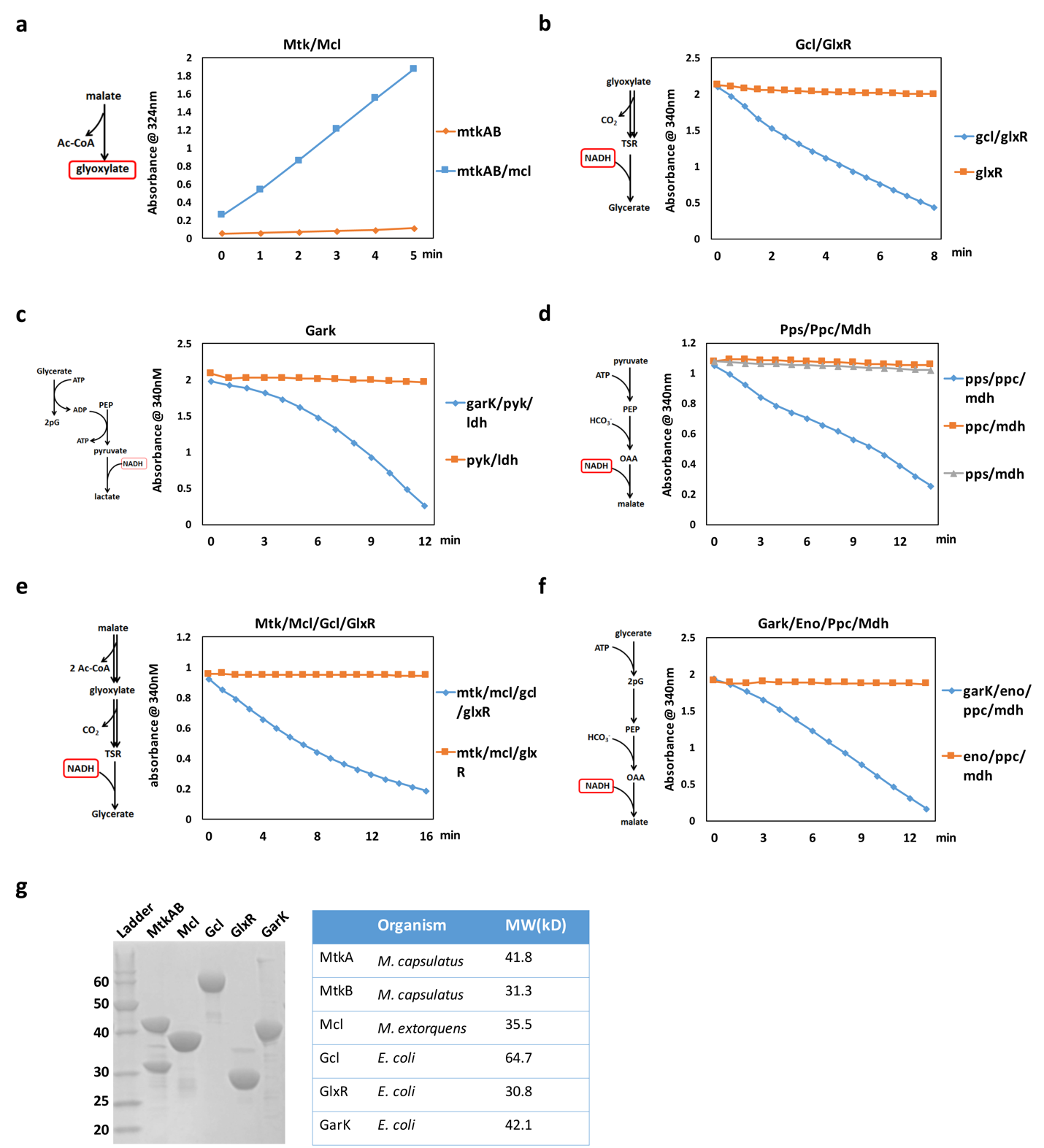


**Supplementary Figure 4. Measurement of enzyme activity *in vitro*.**

(**a**-**f**) Measurements of enzyme activity *in vitro.* Protein amount used for each assay was described in Supplementary Methods.

(**g**). His-tag purification of the MCG enzymes. MW: molecular weight.


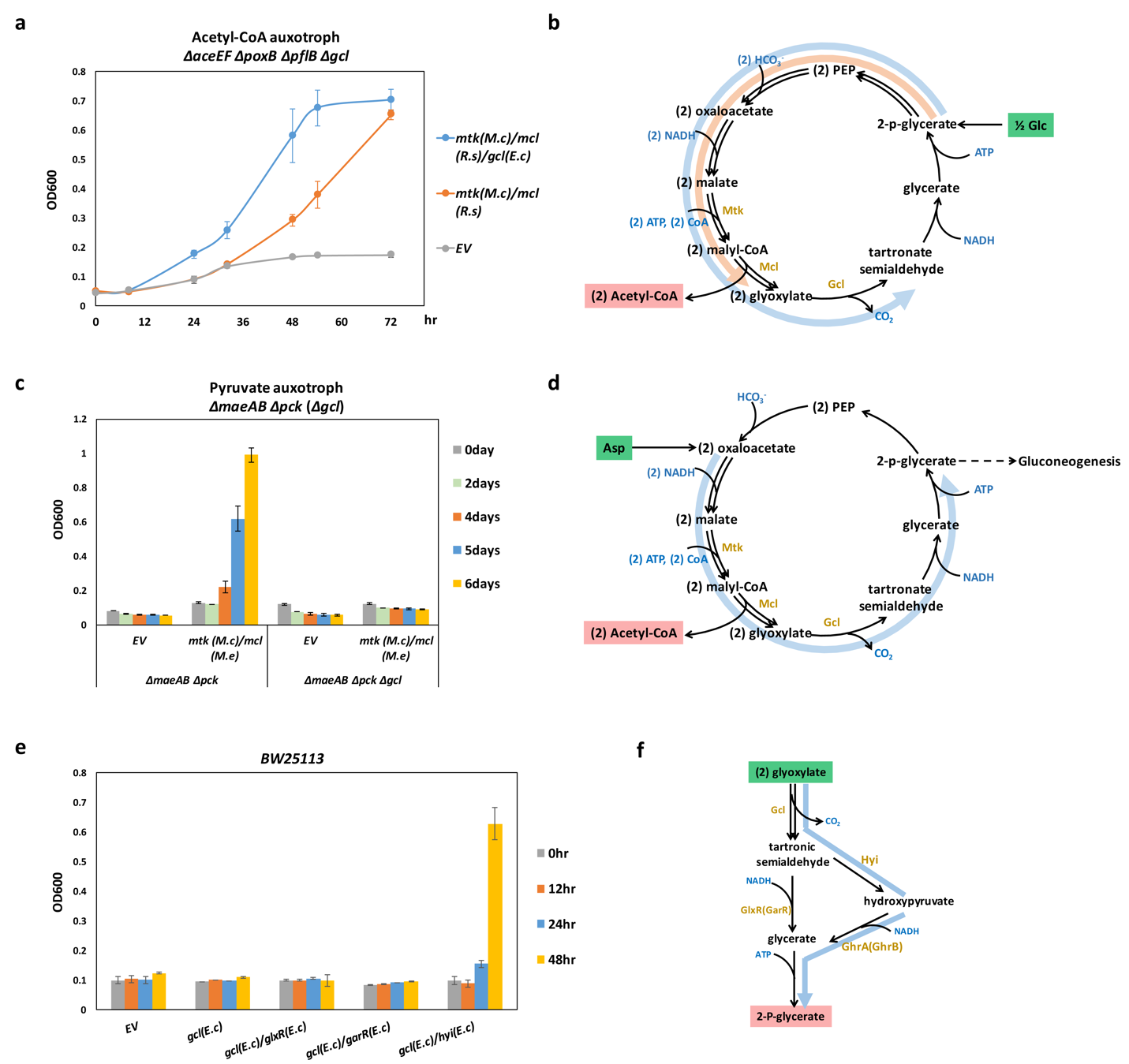


**Supplementary Figure 5. Characterization of the MCG pathway in *E. coli*.**

(**a**-**b**) Expression of *mtk*(*M.c*)/*mcl*(*R.s*) rescued the growth defect of the acetyl-CoA auxotroph *∆aceEF ∆poxB ∆pflB ∆gcl* in minimal medium with 1% glucose addition (**a**) (marked as the orange arrow in **b**). Additional overexpression of *gcl*(*E.c*) could further improve the cell growth*,* which demonstrated the feasibility of the MCG pathway starting from 2-P-glycerate to tartronate semialdehyde (marked as the blue arrow in **b**). EV: empty vector.

(**c**-**d**) Expression of *mtk*(*M.c*)/*mcl*(*M.e*) rescued the growth defect of the pyruvate auxotroph *∆maeAB ∆pck* and supported the strain to grow in minimal medium with 40 mM aspartate as the sole carbon source (**c**). However, with an additional *gcl* knockout, the *E. coli* strain *∆maeAB ∆pck ∆gcl* could not be rescued by *mtk/mcl* expression, which demonstrated the feasibility of the pathway from oxaloacetate to 2-P-glycerate (marked as the blue arrow in **d**).

(**e**-**f**) Overexpression of *gcl*(*E.c*)/*hyi*(*E.c*) allowed the wild type strain to grow in minimal medium with 50 mM glyoxylate as the sole carbon source (**e**), which suggested that the Gcl/Hyi combination played an important role in condensation of glyoxylate to produce glycerate (marked as blue arrow in **f**). Error bars are s.d., n=3.

**
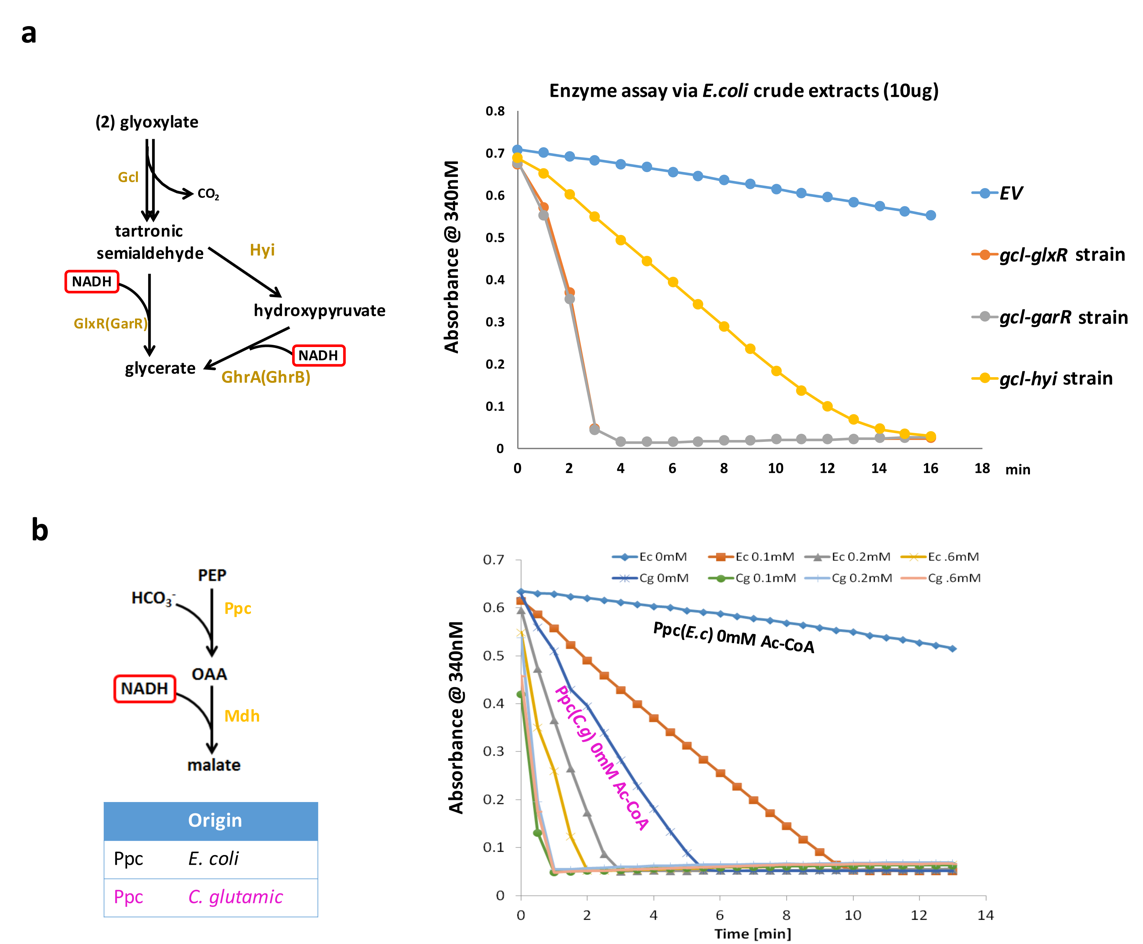
**

**Supplementary Figure 6. Enzyme optimization for construction of the MCG pathway in *E. coli*.**

(**a**) Comparison of enzyme activities among Gcl/GlxR, Gcl/GarR and Gcl/Hyi by measurement of NADH consumption using crude extracts from Supplementary Figure 5e. 10 ug (total protein amount) crude extracts were used. The results showed Gcl/GlxR and Gcl/GarR both displayed higher activities than the Gcl/Hyi combination after IPTG pre-induction. EV: empty vector

(**b**) Ppc(*C. glutamicum*)*,* purified from *E. coli,* displayed much higher carboxylase activity with less acetyl-CoA dependence compared with the one from *E. coli*. For example, Ec 0.1 mM means Ppc from *E. coli* was used in the *in vitro* assay supplemented with 0.1mM acetyl-CoA; Cg 0.1 mM means Ppc from *C. glutamicum* was used in the *in vitro* assay supplemented with 0.1 mM acetyl-CoA.


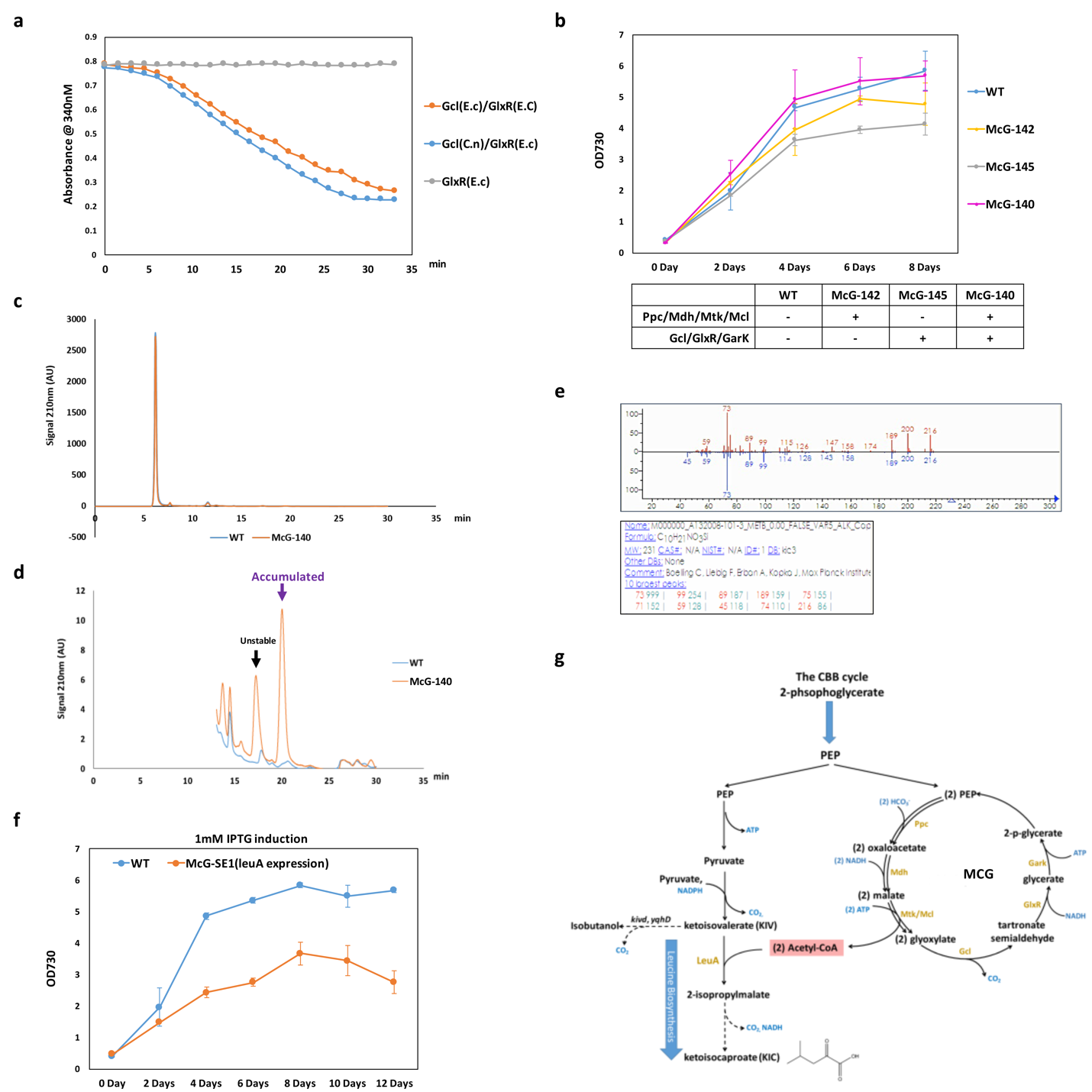


**Supplementary Figure 7. Investigation of effect of the MCG pathway in *S. elongatus*.**

(**a**) Gcl from *Cupriavidus necator* displayed higher activity in glyoxylate condensation compared to the one from *E. coli*.

(**b**) Expression of the complete pathway genes in the strain McG-140 does not negatively affect cell growth compared to wild type, and promoted the growth to saturation faster than the controls expressing the partial pathway genes. Error bars are s.d., n=3.

(**c**-**d**) Two unknown peaks on the chromatogram were found in the supernatant of the McG-140 culture with retention times at 17 min and 20 min. The 17 min peak disappeared after a few days, while the 20 min peak increased by days. (**c**) showed the complete HPLC chromatogram of the wild type and McG-140 sample. (**d**) enlarged the chromatogram with retention time from 14 to 30 mins.

(**e**) GC-MS identification of the KIC production in the McG-140 culture.

(**f**) Overexpression of *leuA*(*E.c*) inhibited cyanobacterial growth. Error bars are s.d., n=3.

(**g**) Illustration of the KIC synthesis pathway in the strain McG-SE7. Enzymes labelled in yellow were expressed in the strain.

**
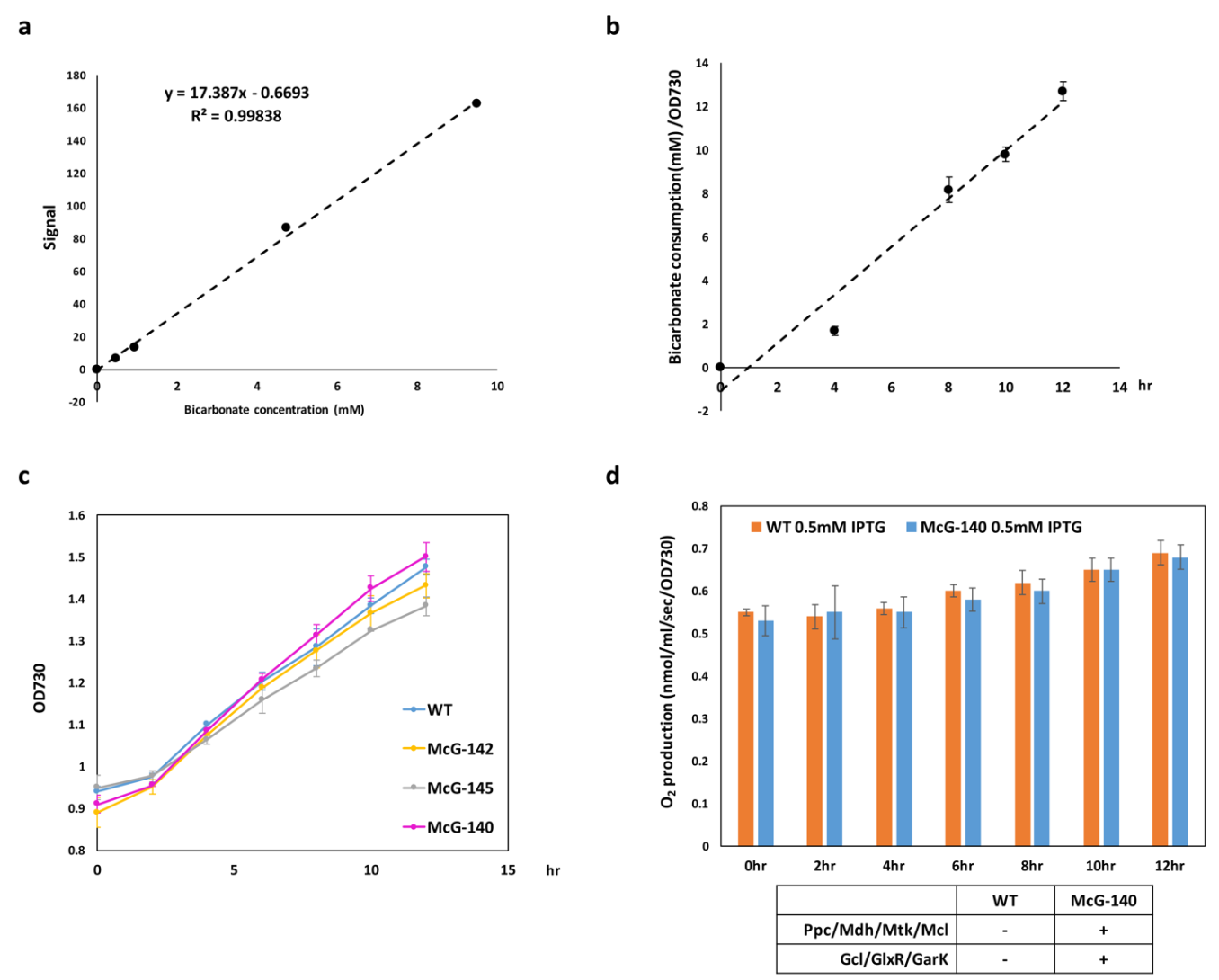
**

**Supplementary Figure 8. Measurement of bicarbonate consumption by NMR spectroscopy.**

(**a**) The standard curve was made by measurement of the NaH^13^CO_3_ reading at various concentrations (0mM, 0.475mM, 0.95mM, 4.75mM and 9.5mM) using NMR spectroscopy.

(**b**) Determination of bicarbonate consumption/OD730 ratio in wild type *S. elongatus*. The wild type *S. elongatus* culture was normalized to OD730 about 1 in fresh BG-11 medium with 50mM 13C-labelled bicarbonate as the sole carbon source under 50μE/s/m^2^ light condition. Bicarbonate consumption and growth were measured in the next 12 hrs.

(**c**) Culture OD measurement for calculation of the bicarbonate assimilation rate in Supplementary Table 7.

(**d**) The strain McG-140 displayed similar level of O_2_ emission as that of wild type.

Error bars are s.d., n=3.

**Supplementary Table 1. Requirements of ATP and NADH equivalents for acetyl-CoA synthesis by various CO_2_ fixation pathways^*^**

| Per Ac-CoA synthesis | Oxygen sensitivity | Status | NADH equiv. | ATP equiv. | The theoretical carbon yield |
| --- | --- | --- | --- | --- | --- |
| The CBB cycle | Tolerant | Natural | 4 | 7 | 66% (Ac-CoA/C3) |
| CBB+MCG | Tolerant | Natural+Synthetic | 4 | 5.5 | 100% (2Ac-CoA/C3+C1) |
| The thaumarchaeal HP/HB cycle^40^ | Tolerant | Natural | 4 | 4 | 100% |
| The crenarchaeal HP/HB cycle^40^ | Tolerant | Natural | 4 | 6 | 100% |
| The 3HP bicycle | Tolerant | Natural | 4 | 7 | 66% (Ac-CoA/C3) |
| The CETCH cycle^46^ | Tolerant | Synthetic | 8 | 2 | 50%(Ac-CoA/2Glyoxylate) |
| The rTCA cycle | Sensitive | Natural | 4 | 2 | 100% |
| The Wood-Ljungdahl pathway | Sensitive | Natural | 4 | 1 | 100% |

^*^The CBB cycle and 3-hydroxypropionate (3HP) bicycle are not evolved for optimal synthesis of acetyl-CoA, the C2 building block. Other natural CO_2_ fixation pathways, such as the reductive tricarboxylic acid (rTCA) cycle, the Wood-Ljungdahl pathway and the HP/HB (hydroxypropionate/hydroxybutyrate) cycle, can synthesize acetyl-CoA from CO_2_ equivalents with 100% carbon yield and lower ATP consumption. However, they have limitations to be constructed in photosynthetic organisms. For example, the rTCA cycle and Woods-Ljungdahl pathway are extremely oxygen sensitivity; the thaumarchaeal HP/HB cycle^40^ includes many enzymatic steps, and some key enzymes have not been well characterized.

**Supplementary Table 2. Thermodynamic calculation of the MCG pathway^*^**

| # | Reactions | ΔrG'm (KJ/mol) | Number | Per Step ΔrG'm |
| --- | --- | --- | --- | --- |
| 1 | CO_2_ + Phosphoenolpyruvate + H_2_O <=> Orthophosphate + Oxaloacetate | -36.7 | 2 | -73.4 |
| 2 | NADH + Oxaloacetate <=> NAD^+^ + (S)-Malate | -30.3 | 2 | -60.6 |
| 3 | ATP + CoA + (S)-Malate <=> ADP + Orthophosphate + Malyl-CoA | -6.7 | 2 | -13.4 |
| 4 | Malyl-CoA <=> Acetyl-CoA + Glyoxylate | -4.2 | 2 | -8.4 |
| 5 | 2 Glyoxylate <=> CO_2_ + Tartronate semialdehyde | -19.6 | 1 | -19.6 |
| 6 | NADH + Tartronate semialdehyde <=> NAD^+^ + D-Glycerate | -33.3 | 1 | -33.3 |
| 7 | ATP + D-Glycerate <=> ADP + 2-Phospho-D-glycerate | -6.7 | 1 | -6.7 |
| 8 | 2-Phospho-D-glycerate <=> Phosphoenolpyruvate + H_2_O | -4.1 | 1 | -4.1 |
| Total | Phosphoenolpyruvate + CO_2_ + H_2_O + 2CoA + 3NADH + 3ATP <=> 2Acetyl-CoA + 3NAD+ 3ADP + 4Orthophosphate | | | **-219.5** |

^*^The reaction Gibbs energy was calculated by eQuilibrator software. ΔrG'm(KJ/mol) is the free Gibbs energy when the reactant concentration was set to be 1 mM under the condition of pH=7 and 0.1 M ionic strength.

**Supplementary Table 3. The specific activity of Mtk/Mcl was associated with the growth-rescuing effect of the strains shown in Figure 2b**

| Gene order | Specific activity  (μmol/mim/mg protein) |
| --- | --- |
| *mtkA(M.c)/mtkB(M.c)/mcl(R.s)* | 0.47 |
| *mtkA(M.c)/mtkB(M.c)/mcl(M.e)* | 1.43 |
| *mtkA(opt)(M.c)/ mtkB(opt)(M.c )/mcl(M.e)* | 1.52 |
| *mtkB(M.c)/mtkA(M.c)/mcl(M.e)* | 2.59 |

**Supplementary Table 4. The specific activity of enzymes used in *in vitro* experiments**

| Enzyme | Organism | Other enzymes added | Substrate(s) | Specific activity (μmol/mim/mg protein) |
| --- | --- | --- | --- | --- |
| Pps | *E. coli* | Ppc, Mdh | 5mM pyruvate | 5.6 |
| Ppc | Microbial | Mdh | 5mM PEP, 5mM NaHCO_3_ | 1.2 |
| Mdh | porcine heart |  | 8mM OAA | 3.1 |
| MtkAB | *M. capsulatus* | Mcl | 10mM malate | 2.7 |
| Mcl | *R. sphaeroides* | MtkAB | 10mM malate | 3.3 |
| Mcl | *M. extorquens* | MtkAB | 10mM malate | 30.3 |
| Gcl | *E. coli* | GlxR | 10mM glyoxylate | 2.8 |
| GlxR | *E. coli* | Gcl | 10mM glyoxylate | 27.8 |
| GarK | *E. coli* | Eno, Ppc,Mdh | 5mM glyceric acid, 10mM NaHCO_3_ | 2.5 |

**Supplementary Table 5. The specific activity of Gcl/GlxR, Gcl/GarR and Gcl/Hyi measured in the strains shown in Supplementary Figure 6a**

| Enzymes | Specific activity  (μmol/mim/mg protein) |
| --- | --- |
| Gcl/GlxR | 1.62 |
| Gcl/GarR | 1.85 |
| Gcl/Hyi | 0.48 |

**Supplementary Table 6. The specific activity of the PPC enzyme from *E. coli* and *C. glutamicum***

| Enzyme | Specific activity  (μmol/mim/mg protein) |
| --- | --- |
| Ppc (*E.c*) | 0.07 |
| Ppc (*C.g*) | 0.86 |
| Ppc (*E.c*) with 0.1mM Ac-CoA | 0.52 |
| Ppc (*C.g*) with 0.1mM Ac-CoA | 3.64 |

**Supplementary Table 7. Calculation of bicarbonate assimilation rate in cyanobacterial strains^*^**

**
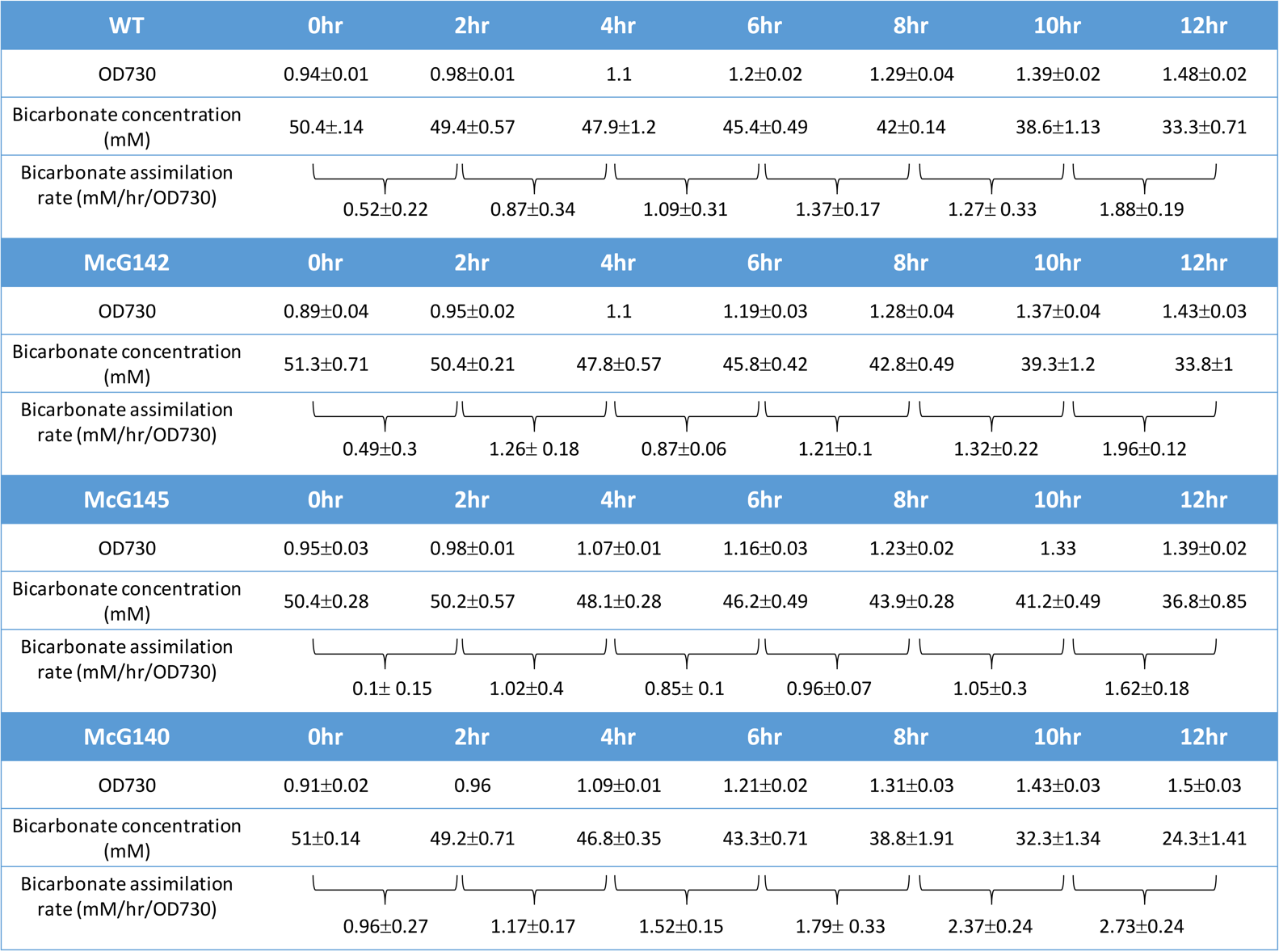
**

^*^The bicarbonate assimilation rate was calculated as: Bicarbonate consumption (mM)/Time interval (2 hr)/Average OD730. 0.5 mM IPTG was used for inducing the expression of the MCG pathway genes in cyanobacterial strains.

**Supplementary Table 8. Additional measurements of bicarbonate consumption by NMR spectroscopy^*^**

**
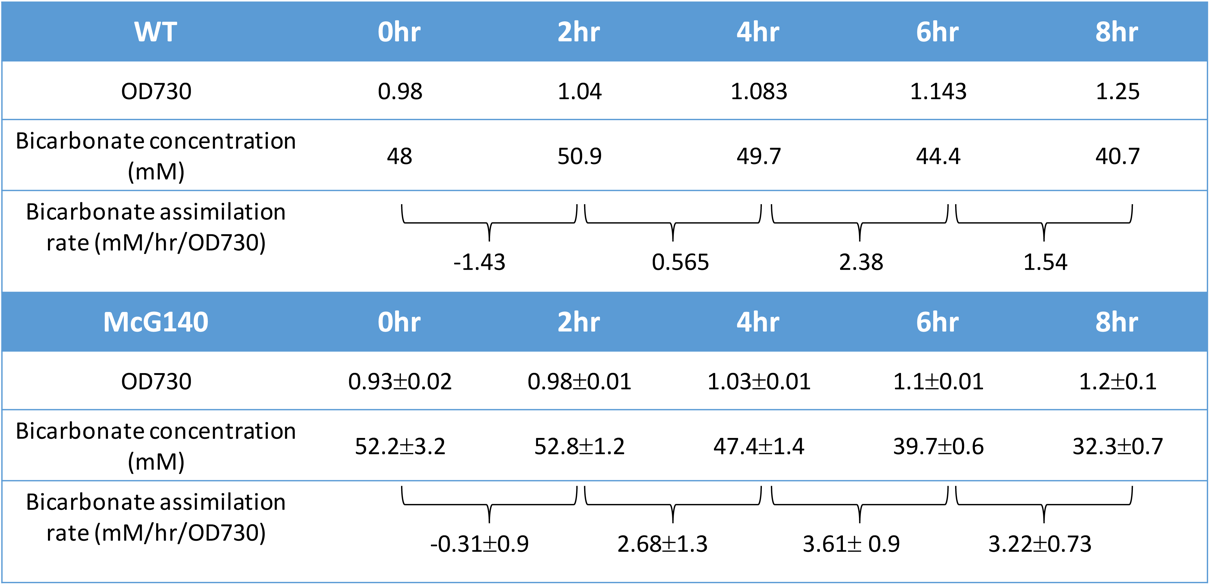
**

^*^Additional experiments were performed independently by another co-author (D.S.C) to measure the bicarbonate consumption. Different from the results shown in Supplementary Table 7, this experiment was performed by using 1 mM IPTG for expression of the MCG pathway genes. Results also showed that the McG-140 strain increased the bicarbonate assimilation rate compared to wild type.

**Supplementary Table 9. Summary of enzymes for the MCG pathway**

| Enzymes | EC # | Gene | Accession ID^*^ | Origin |
| --- | --- | --- | --- | --- |
| Phosphoenolpyruvate carboxylase | 4.1.1.31 | *ppc* | P12880 | *C. glutamicum* |
| Malate dehydrogenase | 1.1.1.37 | *mdh* | P61889 | *E. coli* |
| Malate thiokinase | 6.2.1.9 | *sucCD2* | MCA1740  MCA1741 | *M. capsulatus* |
| Malyl-CoA lyase | 4.1.3.24 | *mcl* | MexAM1_META1p1733 | *M. extorquens* |
| Malyl-CoA lyase | 4.1.3.24 | *mcl1* | RSP_1771 | *R. sphaeroides* |
| Glyoxylate carboligase | 4.1.1.47 | *gcl* | P0AEP7 | *E. coli* |
| Glyoxylate carboligase | 4.1.1.47 | *gcl* | H16_A3598 | *C. necator* |
| Hydroxypyruvate isomerase | 5.3.1.22 | *hyi* | P30147 | *E. coli* |
| Tartronate semialdehyde reductase | 1.1.1.60 | *glxR* | P77161 | *E. coli* |
| Glycerate kinase | 2.7.1.31 | *gark* | P23524 | *E. coli* |
| Enolase | 4.2.1.11 | *eno* | P0A6P9 | *E. coli* |

^*^ All accession IDs are associated with database Biocyc (<https://biocyc.org/>). Sequences can be obtained by searching accession ID and the associated organism in Biocyc.

**Supplementary Table 10. Plasmids and strains used in this study^*^**

| **Experimental models: *E. coli* Strains** | | | |
| --- | --- | --- | --- |
| **Strain** | **Relevant Genotype** | **Plasmid(s)** | **Source** |
| *JCL16* | *rrnBT14 ΔlacZWJ16 hsdR514 ΔaraBADAH33 ΔrhaBADLD78/F’ [traD36 proAB+ lacIqZΔM15]* |  | Atsumi *et al.*, 2008^43^ |
| *BW25113* | *rrnBT14 ΔlacZWJ16 hsdR514 ΔaraBADAH33 ΔrhaBADLD78* |  |  |
| *MC4100* | *F- [araD139]B/r Δ(argF-lac)169* &lambda- e14- flhD5301 Δ(fruK-yeiR)725 (fruA25)‡ relA1 rpsL150(strR) rbsR22 Δ(fimB-fimE)632(::IS1) deoC1* |  |  |
| *SS108* | *∆aceEF ∆poxB ∆pflB* in *JCL16* |  | This work |
| *SS109* | *∆aceEF ∆poxB ∆pflB ∆gcl* in *JCL16* |  | This work |
| *MC4100Δ3* | *∆maeAB ∆pck* in *MC4100* |  | This work |
| *HY41* | *∆aceB ∆glcB ∆frdB ∆ldhA ∆pstG* in *BW25113* | *pYK* | This work |
| *HY42* | *∆aceB ∆glcB ∆frdB ∆ldhA ∆pstG* in *BW25113* | *pHY24* | This work |
| *HY43* | *∆aceB ∆glcB ∆frdB ∆ldhA ∆pstG* in *BW25113* | *pHY25* | This work |
| *HY48* | *∆aceB ∆glcB ∆frdB ∆ldhA ∆pstG* in *BW25113* | *pHY25, pHY28* | This work |
| *HY49* | *∆aceB ∆glcB ∆frdB ∆ldhA ∆pstG* in *BW25113* | *pHY25, pHY29* | This work |
| *HY68* | *∆aceB ∆glcB ∆frdB ∆ldhA ∆pstG* in *BW25113* | *pHY25, pHY29, pHY42* | This work |
| **Plasmids used for *E. coli* strain** | | | |
| **Plasmid** | **Description** | | **Source** |
| *pYK* | *ColA ori; Km^R^* | |  |
| *pHY21* | *ColA ori; Km^R^; PLlacO1:mtkB(M.c)/mtkA(M.c)/mcl(R.s)* | | This work |
| *pHY22* | *ColA ori; Km^R^;PLlacO1:mtkB(M.c)/mtkA(M.c)/ mcl(R.s); PLlacO1:gcl(E.c)* | | This work |
| *pHY24* | *ColA ori; Km^R^; PLlacO1:mtkB(M.c)/mtkA(M.c)/mcl(M.e)* | | This work |
| *pHY25* | *ColA ori; Km^R^; PLlacO1:mtkB(M.c)/mtkA(M.c)/mcl(M.e); PLlacO1:gcl(E.c)/hyi(E.c)* | | This work |
| *pHY28* | *P15A ori; Amp^R^; PLlacO1:garK(E.c)* | | This work |
| *pHY29* | *P15A ori; Amp^R^; PLlacO1:garK(E.c)/mdh(E.c)* | | This work |
| *pHY42* | *PSC101 ori; Spec^R^; PLlacO1:ppc(C.g)* | | This work |
| **Experimental models: *S. elongatus PCC 7942* Strains** | | | |
| **Strain** | **Relevant Genotype** | | **Source** |
| *McG-140* | *pXL142* in *NSI, pXL145* in *NSII* | | This work |
| *McG-142* | *pXL142* in *NSI* | | This work |
| *McG-145* | *pXL145* in *NSII* | | This work |
| *McG-SE1* | *pXL154* in *NSIII* | | This work |
| *McG-SE2* | *pXL19B* in *NSI, pXL154* in *NSIII* | | This work |
| *McG-SE4* | *pXL142* in *NSI, pXL154* in *NSIII* | | This work |
| *McG-SE5* | *pXL145* in *NSII, pXL154* in *NSIII* | | This work |
| *McG-SE7* | *pXL142* in *NSI, pXL145* in *NSII, pXL154 in NSIII* | | This work |
| **Plasmids used for *S. elongatus PCC 7942*** | | | |
| **Plasmid** | **Description** | | **Source** |
| *pXL142* | *ColE1 ori; Spec^R^; PLlacO1:mtkA(M.c)/mtkB(M.c)/mcl(M.e);PLlacO1: ppc(E.c)/mdh(E.c)* | | This work |
| *pXL145* | *ColE1 ori; Km^R^; PTrc:garK(E.c)/glxR(E.c)/gcl(R.e)* | | This work |
| *pXL19B* | *ColE1 ori; Spec^R^; PLlacO1:mtkA(M.c)/mtkB(M.c)/mcl(M.e)* | | This work |
| *pXL154* | *P15A ori; Gent^R^; PTrc:leuA(E.c)* | | This work |

^*^Km^R^: Kanamycin resistance; Amp^R^: Ampicillin resistance; Spec^R^: Spectinomycin resistance; Gent^R^: Gentamycin resistance. RBS (*E. coli*): 5’- AGGAGATATACC-3’; RBS (*S. elongatus*): 5’- AGGAGAAAGGTACC-3’.

**Supplementary Table 11. Primers used in this study**

| Primer name | Sequence |
| --- | --- |
| *mtkA(M.c) F* | 5’-GATTCTGAAGGAGATATACCATGAATATCCATGAGTACCA-3’ |
| *mtkA(M.c) R* | 5’-GCTCATGGTATATCTCCTTTATCCCTTGACGATGGCGA-3’ |
| *mtkB(M.c) F* | 5’-AGAGGAGATATACCATGAGCGTATTCGTTAACAAG-3’ |
| *mtkB(M.c) R* | 5’-ATATTCATGGTATATCTCCTTCAGAATCTGATTCCGTGTT-3’ |
| *mcl(M.e) F* | 5’-CAAGGGATAAAGGAGATATACCATGAGCTTCACCCTGATCCA-3’ |
| *mcl(M.e) R* | 5’-CAAGCTTCTCGAGTTACTTTCCGCCCATCGCGT-3’ |
| *gcl(E.c) F* | 5’-AGAGGAGATATACCATGGCAAAAATGAGAGCCGT-3’ |
| *gcl(E.c) R* | 5’-ACGTAACATGGTATATCTCCTTTATTCATAGTGCATGAAGCA-3’ |
| *gcl(C.n) F* | 5’-AGAGGAGATATACCATGGCAAAGATGAGAGCAAT-3’ |
| *gcl(C.n) R* | 5’-GAAACGTAACATGGTATATCTCCTTTACGCCGTTTCCACTTCTT-3’ |
| *glxR(E.c) F* | 5’-GAATAAAGGAGATATACCATGAAACTGGGATTTATTGGCT-3’ |
| *glxR(E.c) R* | 5’-CAAGCTTCTCGAGTCAGGCCAGTTTATGGTTAG-3’ |
| *hyi(E.c) F* | 5’-CTATGAATAAAGGAGATATACCATGTTACGTTTCTCTGCTAA-3’ |
| *hyi(E.c) R* | 5’-CAAGCTTCTCGAGTTAACGGTACGGATCCATCC-3’ |
| *gark(E.c) F* | 5’-GGAGATATACCATGGCGTATTGCAATCCGGG-3’ |
| *gark(E.c) R* | 5’-ATCGATACCGTCGACTCACCCCGCGTTGCGCATTC-3’ |
| *ppc(C.g) F* | 5’-GAGGAGATATACCATGACTGATTTTTTACGCGAT-3’ |
| *ppc(C.g) R* | 5’-ATCAAGCTTCTCGAGCTAGCCGGAGTTGCGCAGCG-3’ |
| *ppc(E.c) F* | 5’-GAGGAGATATACCATGAACGAACAATATTCCGC-3’ |
| *ppc(E.c) R* | 5’-CAAGCTTCTCGAGTTAGCCGGTATTACGCATACCT-3’ |
| *mdh(E.c) F* | 5’-GAGGAGATATACCATGAAAGTCGCAGTCCTCGG-3’ |
| *mdh(E.c) R* | 5’-GCCTCGTGATACGCCTTACTTATTAACGAACTCTT-3’ |
| *leuA(E.c) F* | 5’-GAGGAGATATACCATGAGCCAGCAAGTCATTAT-3’ |
| *leuA(E.c) R* | 5’-CGATACCGTCGACTCACACGGTTTCCTTGTTGTT-3’ |
